# Surface Properties of Graffiti Coatings on Sensitive Surfaces Concerning Their Removal with Formulations Based on the Amino-Acid-Type Surfactants

**DOI:** 10.3390/molecules28041986

**Published:** 2023-02-20

**Authors:** Marcin Bartman, Sebastian Balicki, Lucyna Hołysz, Kazimiera A. Wilk

**Affiliations:** 1 Department of Engineering and Technology of Chemical Processes, Wrocław University of Science and Technology, Wybrzeże Wyspiańskiego 27, 50-370 Wrocław, Poland; 2 Department of Interfacial Phenomena, Institute of Chemical Sciences, Faculty of Chemistry, Maria Curie-Skłodowska University in Lublin, M. Curie-Skłodowska sq. 3, 20-031 Lublin, Poland

**Keywords:** ecological graffiti remover, sensitive surfaces, amino-acid-type surfactants, surface roughness, wettability, contact angle, surface free energy, work of adhesion, work of spreading

## Abstract

Water-in-oil (w/o) nanoemulsions stabilized with amino acid surfactants (AAS) are one example of nanotechnology detergents of the “brush on, wipe off”-type for removing graffiti coatings from different sensitive surfaces. The high-pressure homogenization (HPH) process was used to obtain the nanostructured fluids (NSFs), including the non-toxic and eco-friendly components such as AAS, esterified vegetable oils, and ethyl lactate. The most effective NSF detergent was determined by response surface methodology (RSM) optimization. Afterwards, several surface properties, i.e., topography, wettability, surface free energy, and the work of water adhesion to surfaces before and after their coverage with the black graffiti paint, as well as after the removal of the paint layers by the eco-remover, were determined. It was found that the removal of graffiti with the use of the NSF detergent is more dependent on the energetic properties and microporous structure of the paint coatings than on the properties of the substrates on which the layers were deposited. The use of NSFs and knowledge of the surface properties could enable the development of versatile detergents that would remove unwanted contamination from various surfaces easily and in a controlled way.

## 1. Introduction

Due to their durability, attractiveness, and availability, materials such as glass, aluminum alloys, stone, and marble are extensively utilized in architecture, in both public and private buildings. Nevertheless, because of their popularity, vandalism is common on the surfaces of these materials. For the most part, this means that the objects lose their aesthetic value, and after extended exposure to physical factors, their surfaces can experience a shift in their own qualities [1,2]. Traditionally, chemicals, physical methods, and more recently, biological approaches have been used to strip off undesired graffiti coatings [3,4,5,6,7]. Furthermore, chemical cleaning in conjunction with mechanical action is by far the most common method of removing graffiti from a surface. However, the fundamental issue with these actions is the threat to the surface integrity by the visible alteration of the substrate color, the removal of some of the mineral particles, and the formation of gaps on the surface, considerably altering the surface roughness [8]. The coatings durability depends upon the adhesion strength paint/surface [9]. Strong adhesion allows the materials to function properly as coatings on the substrates [10,11]. In many cases, adhesion is a desirable phenomenon, but on the other hand, the adhesion of food products to the surface of oil contaminants or paints on various surfaces (graffiti) is unfavorable because it increases the surface cleaning cost [3].

Adhesion is a phenomenon involving the attachment of two surfaces of different phases as a result of the action of chemical and/or physical adhesive forces such as van der Waals (dispersion, dipole−dipole, dipole-induced dipole), electron–donor and electron–acceptor (including hydrogen bonding), *π*-electrons and electrostatic forces. The DLVO theory describes the interactions of forces acting on a surface [12,13]. The work of adhesion, whose value is a measure of the intermolecular attraction between two distinct phases, may be used to characterize the strength of the contacts between two surfaces. In the case of a solid and a liquid, the work of adhesion ($W_{A}$) can be expressed by the relation [12,14]:

(1) $$W_{A}=\gamma_{SV}+\gamma_{LV}-\gamma_{SL}$$

where $\gamma_{SV}$, $\gamma_{LV}$ and $\gamma_{SL}$ are the liquid–vapor, solid–vapor, and solid−liquid interfacial surface free energies, respectively.

In the absence of any chemical interactions, the adhesion strength is determined by the molecular physical interactions. The surface roughness also plays an important role [13,15,16,17,18]. The adhesion of a liquid to a solid surface is commonly investigated via the contact angle measurements, and then by the determination of the solid surface free energy and the thermodynamic work of adhesion. In the case of a smooth, homogeneous, and isotropic solid surface, the contact angle ($\theta_{Y}$) is described by the Young’s equation [19,20]:

(2) $$cos\theta_{Y}=\frac{\gamma_{SV}-\gamma_{SL}}{\gamma_{LV}}$$

In the case of rough and/or heterogeneous solid surfaces, the contact angle is described by the Wenzel equation on the rough surface and the Cassie–Baxter equation on the heterogeneous surface [21]. The Wenzel equation is given by [22]:

(3) $$cos\theta_{W}=r\frac{\gamma_{SV}-\gamma_{SL}}{\gamma_{LV}}=rcos\theta_{Y}$$

where *r* is the roughness parameter that expresses the ratio of the true solid surface to its horizontal projection.

As the r value is always greater than 1, the surface roughness enhances the hydrophilicity of the wetted surfaces or the hydrophobicity of the non-wettable surfaces. The Cassie–Baxter equation describes the contact angle of a liquid droplet that is in contact partly with the surface of the solid and partly with the air in its cavities [23]:

(4) $$cos\theta_{CB}=\frac{f_{1}\left(\gamma_{SV}-\gamma_{SL}\right)-f_{2}\gamma_{LV}}{\gamma_{LV}}=f_{1}cos\theta_{Y}+f_{2}$$

where *f_1_* and *f_2_* are the coefficients representing the fractions of the liquid droplet contact area at the solid–liquid and liquid–air interfaces, respectively.

Having determined the contact angle, it is possible to calculate the thermodynamic work of adhesion [14]:

(5) $$W_{A}=\gamma_{LV}\;\left(1+cos\theta \right)$$

The combination of wettability and adhesion force is expressed by the work of spreading $W_{S}$, which can be derived from the work of adhesion $W_{A}$ and the work of cohesion $W_{C}$:

(6) $$W_{S}=W_{A}-W_{C}=\gamma_{LV}\;\left(cos\theta -1\right)$$

where $W_{C}=2\gamma_{LV}$.

This allows characterization of the competition between the liquid/solid adhesions with a variety of liquids or substrates differing in their polarities [24]. $W_{S}$ is the thermodynamic quantity that relates the wettability to the mechanical strength of adhesion. It enables the characterization of the competition between the solid–liquid adhesions with different liquids [25].

For the determination of the solid surface free energy, there are different theoretical approaches to the nature of the interfacial interactions [26,27,28]. One of the approaches uses the advancing ($\theta_{a}$) and receding ($\theta_{r}$) contact angles of only one liquid [29,30,31]. The advancing contact angle is measured for a liquid drop whose volume increases upon the contact with the solid surface. After reducing the droplet volume for almost every solid/liquid/gas system, the three-phase contact line is receded, and the receding contact angle under the equilibrium conditions is smaller than the advancing contact angle. The receding contact angle shows the strength of liquid/solid adhesion [32,33,34]. The difference between these two contact angles is called the hysteresis of the contact angle, and its origin, among others, is due to the surface roughness, the chemical heterogeneity of solids [32,35] and/or the liquid film left behind the retreating droplet. Chibowski [29,30,31] proposed the quantitative interpretation of the contact angle hysteresis (CAH), assuming that the difference between the advancing and receding contact angles results from the presence of a liquid film behind the droplet. Hence, the total surface free energy of a solid ($\gamma_{S}$) can be obtained from the three measurable parameters: the probe liquid surface tension ($\gamma_{LV}$) and the advancing and receding contact angles measured on the investigated solid surface.

(7) $$\gamma_{S}=\frac{\gamma_{LV}{\left(1+cos\theta_{a}\right)}^{2}}{2+cos\theta_{r}+cos\theta_{a}}$$

The real solid surfaces are more or less rough; therefore, the surface free energies calculated from Equation (7) should be considered apparent. Nevertheless, changes in the surface free energy due to a given surface treatment provide interesting information about the paint adhesion to the surface and its removal.

The aim of this study was to determine the surface properties (topography, wettability, surface free energy, and work of adhesion) of sensitive surfaces, i.e., different commercial decorative tiles (glass, aluminum, stone, and marble) before and after the black paint treatment and after the selective removal of the graffiti coatings from these surfaces using the ecological graffiti remover. For this purpose, water-in-oil (w/o) nanoemulsions were developed, which are nanotechnological “brush on, wipe off” detergents. To maintain the integrity of the nanoemulsion formed during the high-pressure homogenization, a variety of commercially available, mild, and environmentally friendly anionic surfactants of the amino-acid-type (AAS) were chosen and utilized (their structures and abbreviations are shown in Table 1). Currently, there are only a few studies [36,37,38,39,40] that examine the possibility of using AAS to manufacture nanostructured fluids (NFs) to be used as graffiti cleaners. One of the criteria for selecting a suitable AAS was the presence of a glycine molecule or its direct analog in the structure of the amino acid composing the AAS. The D-optimal design model followed by the response surface methodology (RSM) was applied to select an optimal w/o nanoemulsion, which was then used as a “brush on, wipe off” nanotechnological detergent for the selective removal of graffiti coatings and verification of the effect of this type of detergent on the surface properties of sensitive substrates. In fact, the physical and/or chemical adhesion of nanostructural fluid to the surface of graffiti paints is crucial for their effective removal [41,42,43,44,45]. Therefore, in addition to assessing the spreading effectiveness of the NFs fabricated by our team on the surface of selected sensitive surfaces coated with the black paint, experiments were also performed to monitor changes in the wettability of the graffiti-painted surface, taking the simplicity of removal into account.

## 2. Results and Discussion

Currently, there is a significant demand for “brushed on, wiped off” nanodetergents to remove undesirable paint coats due to the growing awareness of the need to save water and reduce dependence on petrochemical raw materials. In this paper, we describe the water-in-oil (w/o) nanoemulsions stabilized with the amino acid surfactants (AAS), formed due to the process of high-pressure homogenization (HPH), resulting in the formation of w/o nanoemulsion droplets with the size in the range of approximately 200–500 nm, as part of our ongoing research on the development of this class of nanodetergents [42].

Quick and safe removal of graffiti coatings without the need for specialized equipment and excessive amounts of energy and water is an integral part of the technology of cleaning sensitive surfaces with NSF detergents. In Section 3.1, the proposed AAS-based NSF detergents were discussed in detail. The previous research [42] highlighted the importance of using nonionic surfactants of the alkyl polyglucosides (APG) type in the formulation of the eco-graffiti remover, which effectively reduced surface tension [47,48]. Surfactants play an important role because they initiate the process of reducing the polymer (paint)–surface and polymer–polymer interfacial tension, thus promoting the detachment of the film from the substrate. The main function of the surfactant is to weaken the chemical and/or physical forces of all paint components adhered to the substrate. In addition, the NSF developed by our group can go through the paint porous surface and inside, where it can start dissolving the polymer [42].

However, it is worth mentioning that the film-forming coats of polymers typically employed to preserve, maintain, and renew the surfaces of the works of art or the monuments against the deterioration caused by weather conditions are removed using the nanostructured liquids in the extensive studies by the other authors [43,44,45,49,50,51,52]. The mechanism for removing this type of coating, so-called “dewetting”, has been well-described by Baglioni et al. [51,52].

### 2.1. Preparation, Characterization and RSM Optimization of w/o Nanoemulsions

**Figure 1:**
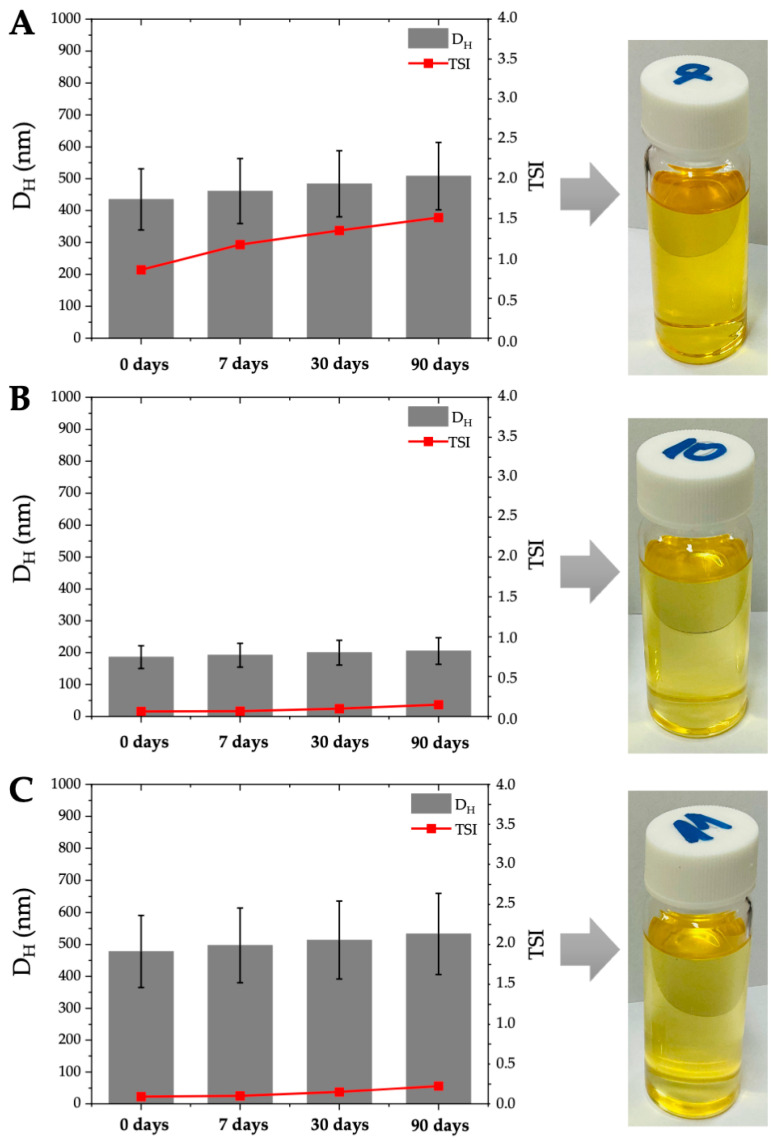
Changes in the key parameters (DH and TSI) to characterize the stability of nanoemulsions during the 3-month storage period. (**A**) NE No. 7. (**B**) NE No. 10. (**C**) NE No. 11.

Our study showed that, using the high-pressure homogenization technology [53,54], it is possible to produce water-in-oil nanoemulsions stabilized by the amino-acid-type surfactants. As a result, transparent, yellowish, and homogeneous w/o nanoemulsions were obtained (as shown in Figure 1) which were then subjected to the long-term thermokinetic stability study that lasted at least 90 days and carried out at room temperature (20 ± 5°C). To observe changes in the droplets size (D_H_), the polydispersity index (PDI) and the physical stability of the preparation, as determined by the Turbiscan stability index (TSI), were measured in each stage of the technological manufacturing process [53,55]. As expected, using the non-pressure homogenization technology and various types of AAS as the emulsion stabilizers, the pre-emulsion that had the average droplets size between 1.1 and 11.0 μm and the polydispersity index ranging from 0.024 to 0.140 were produced (see Table S1), characterized by the short-term thermokinetic stability TSI (60 min) < 5.1. Surprisingly, the NE formulations based on the amino acid surfactant SCG showed good thermokinetic stability during the storage for 90 days (TSI (90 days) <9.0). Then, the obtained pre-emulsions were subjected to the high-pressure homogenization process at the working pressure of 100 MPa or 150 MPa. As a result, at the working pressure of P = 100 MPA the pre-emulsions decreased their average droplets size by approximately 35–49%, and at the pressure of P = 150 MPA by approximately 75–86%; furthermore, their physical stability, defined by the TSI parameter, was significantly improved (see Table S2 and Table 2). As a result of the high pressure at P = 150 MPa, nanoemulsion structures were produced for NE No. 7, NE No. 10 and NE No. 11 at the amino acid surfactant concentrations of 0.05 mol/dm^3^ SCMT and 0.05 and 0.075 mol/dm^3^ SCG. The obtained colloidal nanoemulsion structures were characterized by the average droplets size in the range of 186–478 nm while maintaining a similar range of PDI <0.1 (PDI = 0.037–0.049) which proves very good homogeneity of the obtained nanoemulsion systems (see Table 2). Our research has also proven that the obtained narrow distributions of monodisperse water droplets in the oil phase were very thermokinetically stable during long-term storage at room temperature. As a result, only slight changes in the TSI parameter (TSI (0 days) = 0.06–0.86 and TSI (90 days) = 0.15–1.51) and the average droplets size (D_H_ (0 days) = 186–478 nm and D_H_ (90 days) = 205–533 nm) were observed for 90 days (see Table 2). In addition, very time-stable colloidal systems were obtained, for which typical aggregation phenomena such as coalescence, sedimentation, or flocculation of droplets were not observed [55]. Surprisingly, the NE formulations based on SLG and SCCG, i.e., amino acid surfactants based on glutamine, were not able to form nanoemulsion structures. At the same time, it was found that all preparations based on SCCG exhibited only short-term thermokinetic stability, while in the case of long-term storage at room temperature, they were characterized by very high instability of the emulsion system (TSI (7 days) >30), which was visible in the form of coalescence and the sedimentation of droplets.

The amino acid surfactants used in our study, SCMT and SCG, characterized by the HLB of 6.1–7.5 and the surface tension ($\gamma_{LV}$) of 26.4–35.3 mN/m, tended to produce more stable colloidal systems in their emulsions or nanoemulsions than SLG (HLB = 9.7, $\gamma_{LV}\;$= 35.7 mN/m) or SCCG (HLB = 8.3, $\gamma_{LV}\;$= 29.9 mN/m) (see Table 1). Therefore, the amino acids comprising the AAS structure [56] were of key importance to producing time-stable colloidal systems, such as water droplets dispersed in the oil phase produced in the process of high-energy homogenization. During the high-pressure homogenization process, AAS are adsorbed at the oil–water interface, reducing the interfacial energy, and providing a mechanical barrier to the coalescence processes or other physical destabilization processes of the nanoemulsion [57,58,59]. The obtained results indicated that the simpler the structure of the amino acid was (in our study this was an amino acid based on glycine), the easier it was to produce a stable long-term structure of the nanoemulsive fluid. In general, the results obtained during the long-term stability studies of w/o nanoemulsions prepared using the HPH technology indicate that the obtained SCG-based NSFs were long-term thermokinetically stable, which allows them to be commercially used as the “brush on, wipe off” detergents.

The optimal conditions of the HPH process for the fabrication of AAS-based NSFs, together with the desirable amino acid surfactants and their concentrations, were studied using the quadratic D-optimal design model. The existing relationship between the input variables, i.e., AAS concentration (A), HPH pressure applied (B), and type of AAS (C) and the response variables, i.e., particle diameter (Y_1_), PDI (Y_2_), TSI after 0 days (Y_3_), TSI after 7 days (Y_4_), and TSI after 30 days (Y_5_), was evaluated. The randomized 38-run experimental matrix of the D-optimal design, with the numerical values of the corresponding independent and dependent variables, is shown in Table S4. The 3D response surface plots, based on the multiple regression model (D-optimal), graphically represent the potential interactions between the process and response factors (see Figures S2 and S3). The main goal of the optimization was to determine the conditions that allow for the creation of the AAS-based NSF system with great kinetic stability (minimized TSI values), uniform monodispersity (PDI as low as possible), an average particle size within the nanoscale (500 nm and less), and preserved effectiveness in removing unwanted paint coatings from the sensitive surfaces. The results of the D-optimal design evaluation allowed to describe the most desirable fabrication parameters. The ANOVA metrics, followed by multiple regression analysis (see Text S1 and Table S5), pointed out that the most efficient parameters for production of AAS NSFs are in the 3D response surfaces (Figures S2–S5) within a combination of the lowest concentration of AAS (A), the highest homogenization pressure (B), and the amino acid surfactants exhibiting lower HLB values: SCMT and SCG (C). The discussed surfaces correspond to the interactions between factors A and B (in the function of the factor C) and the dependent variables Y_1_–Y_5_. In general, AAS with lower HLB, especially the one derived from glycine (SCG), followed by its lower concentration in the NSF system, noticeably increased the quality of fabricated w/o nanoemulsions, i.e., they exhibited the smallest droplet diameter, highly uniform dispersity (the smaller PDI), and great kinetic stability (TSI values in the range of 0.06–1.51). On the other hand, the homogenization pressure (B) also had a strong positive influence on the quality of the prepared NSFs, where within the highest-pressure values it was possible to achieve the production of greatly stable w/o nanoemulsions. To summarize, the D-optimal design optimization pointed out that SLG- and SCCG-based NSFs should not be considered in further analyses due to their poor stability and micrometric size of particles. Secondly, the combination of the highest pressure of HPH and the lowest concentrations of AAS gave satisfactory formulations. As a conclusion, the SCG-based system with the concentration of AAS between 0.05 and 0.075 mol/dm^3^ was suggested by the D-optimal model optimization, which refers to the formulations NE No. 10 and No. 11. The characteristics of NE No. 10 and No. 11, with predicted values from the RSM model and actual values of response factors, followed by the desirability function measure [41,42] are presented in Table 3. However, of the two candidates, NE No. 10 turned out to be the optimal one, due to its approximately 50% better stability index (up to 90 days of storage), the average particle diameter of less than 220 nm (vs. 478 nm), and noticeably smaller PDI values. Therefore, formulation NE No. 10 was subjected to further studies as described in the following sections.

### 2.2. Surface Properties of Sensitive Surfaces

**Figure 2:**
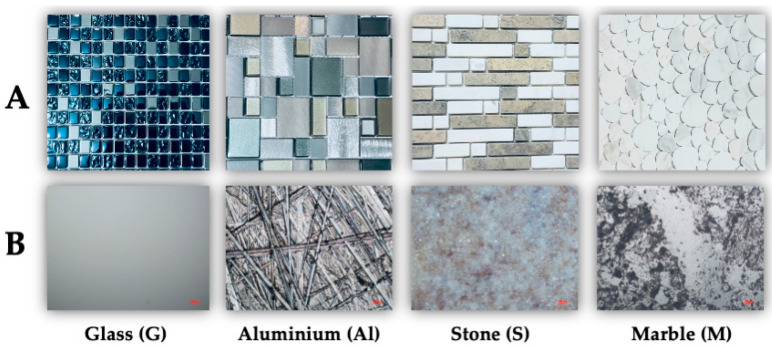
Photographs of the reference sensitive surfaces available on the commercial market (**A**). Images of the reference sensitive surfaces at 40× magnification (**B**).

Graffiti can be seen on the facades of both private and public buildings, underground passages, viaducts, and railway stations. Inscriptions and signs are often painted with easily available spray paints. The durability of such coatings depends on the surface properties of the substrates on which they were used, i.e., the type of substrate, its surface layer structure, and the adhesion strength of oil or acrylic paints. Considering the practical aspects related to the graffiti coatings removal, various materials that are often used in architecture were chosen to study considering their durability, aesthetics, availability, and ease of implementation. For these purposes, sensitive surfaces obtained from man-made materials (glass (G), aluminum alloy (Al)) and natural materials (stone-travertine (S) as well as marble (M)) were used (Figure 2). These surfaces are applied as decorative wall tiles. Evaluation of the removal of the Montana paint coating from the sensitive surface substrates was made using wettability measurements of the advancing and receding water contact angles, determination of adhesion work and surface free energy, as well as surface roughness parameters.

#### 2.2.1. Surface Structure Analysis

Surface roughness is an important parameter affecting the bond strength between the paint coatings and the substrates [60]. In view of the mechanical theory of adhesion, an increase in the microroughness has a beneficial effect on increasing the actual surface area at the coating–substrate interface. The paint fills micro-irregularities and, after hardening creates mechanical anchors, promoting its bond strength. Surface topography plays an important role in the study of surface properties of solids because roughness can affect wettability [61,62,63,64]. 

Therefore, optical profilometry was used to obtain information about the structure of the tested sensitive surfaces before and after the black paint treatment and selective removal of the graffiti coatings from these surfaces using an ecological graffiti remover. In addition, the effect of the ecological removal on the clean bare surfaces cleaning and their surface properties was investigated.

**Figure 3:**
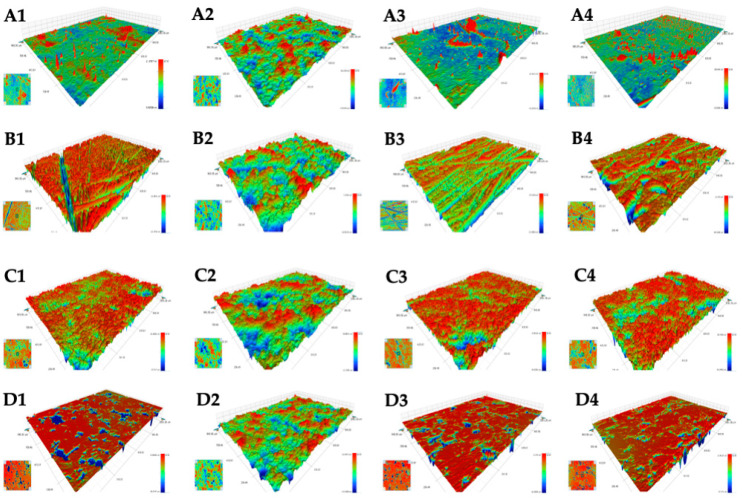
Optical profilometer images of 0.94 × 1.3 mm^2^ surface areas of **A**—glass (G), **B**—aluminum (Al), **C**—stone (S), **D**—marble (M), 1—bare surface sample, 2—surface covered with paint, 3—surface after removal paint coating with NE No. 10 removal, and 4—bare surface after cleaning with NE No. 10.

Figure 3 shows the 3D and 2D images of the tested sensitive surfaces. On each plate, an area of 0.94 × 1.3 mm^2^ was scanned in three places, and the images corresponding to the average value of the roughness parameters are shown.

Analyzing the surface images, the amplitude parameters of the surface roughness were determined using the Vision 4.20 Veeco image processing software. The arithmetic means of the absolute height ($R_{a}$), the root-mean-square value (rms) of the ordinate values within the sampling area ($R_{q}$) and the differences between the peak values ($R_{t}$) are summarized in Table 4. These parameters were calculated over the entire measured array, and they quantitatively characterized the roughness of the surfaces [65,66].

Comparing the roughness parameters of the bare sensitive surfaces, glass with a rms value of 0.62 nm and the average roughness $R_{a}$ of 0.37 nm possessed the smoothest surface which was similar to the values obtained by other authors [67,68,69]. Significantly greater roughness occurred on the other three surfaces, with the surface roughness parameters being virtually the same for the bare surfaces of aluminum alloy (Al-B) and stone (S-B). Nevertheless, on the aluminum alloy, they were artificially produced while the stone was composed of minerals, empty spaces, pores, or fissures whose volume and distribution significantly affect the stone’s behavior [70].

**Figure 4:**
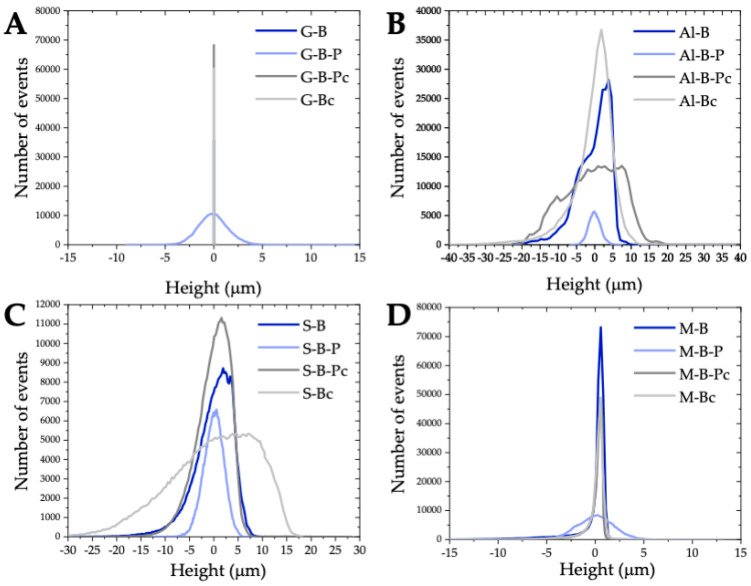
Roughness height distribution on the surfaces of glass (**A**), aluminum (**B**), stone (**C**) and marble (**D**) of the dimensions of 0.94 × 1.3 mm^2^.

The roughness height distribution on these surfaces differed slightly as seen in Figure 4B,C. On the Al-B surface, there were 52.5% hills and 32.5% holes up to 5 mm, whereas there were 50.6% hills and 36.4% holes on the S-B surface. The original Al alloys roughness was between 0.1 and 1 µm [71,72,73,74], depending on the surface composition. Several chemical and physical methods were used to increase the surface roughness of aluminum alloys and enhance their wetting properties. Torrisi et al. [72] used six methods of surface treatment to change the aluminum structure (polishing, sanding, acid etching, laser ablation, ion implantation and nanoparticle deposition). Polishing the surface with the abrasive micro- to submicro-silicate grains dispersed in the solution resulted in the mirror surface. In turn, such surface treatments of aluminum resulted in the decrease in the $R_{a}$ value from 0.1 to 0.028 µm and a slight increase in the water contact angle from 95 to 99°. On the other hand, sandblasting, consisting in spraying SiO_2_ particles with a diameter < 10 µm in air onto the Al surface, caused a significant increase in roughness to 3 µm and 4.5 µm after 30 s and 3 min of treatment, respectively. In this case, an increase in the hydrophobicity of the surface was observed, appearing as a decrease in the water contact angles to 83° and 63°, respectively. It was postulated that on the modified Al surfaces, the water contact angle was practically proportional to the average roughness which can be expressed by the almost linear relationship $\theta_{y}=14R\;\left(µm\right)+100$ [72]. Kubiak et al. [62] investigated the effect of surface roughness on the wettability of engineering materials (aluminum, titanium, steel, copper alloys, ceramic and PMMA). In the case of the A7064 aluminum alloy, a significant effect of the surface roughness on the contact angle of water was found. On the surface with an average roughness of 0.22 μm, the water contact angle was 78.9° whereas at $R_{a}$ = 3.48 μm it was already 86.9°. 

Natural stones comprised two other substrates that were covered with the graffiti. For ages, they have been used in the construction of various buildings in all architectural forms. Currently architects turn back to the use of natural stone for the interior design, e.g., wall cladding. As already mentioned, tiles made of natural stone (travertine) and marble were used in our research. They belong to limestone sedimentary rocks, with travertine being more porous. In the past travertines were applied as both a building material and decorative stones. The natural stones are mostly prepared as tiles or slabs for the internal use and for the terraces. Travertine is always offered in the “open-pore” or “trowelled version”. Marble is an extremely common decorative stone willingly applied for architectural and sculptural purposes. Its advantage is that it is relatively easy to grind and polish, especially in the process of finishing glossy decorative materials. Compared to the tiles made of stone, its surface is smoother. The average roughness on the bare marble surface was 1.12 ± 0.2 µm, and approximately 78% of its surface was covered with hills, up to 1 µm (Figure 4). In the case of the stone (S-B), the $R_{a}$ was 3.78 ± 1.4 μm with 51.6% of its surface being holes up to 5.3 μm and hills up (37%) to 5.9 μm.

Çıra et al. [75] studied the effects of the physico-mechanical and mineralogical properties as well as the chemical contents of four limestones on their final glossiness and roughness values. They carried out polishing tests which allowed to determine the effect of material properties and these parameters on the quality of their surface. Their study revealed that coarse abrasives (from 60 to 320) had a more pronounced effect on the decrease in the surface roughness (from 2.4 to 0.3 μm) without a significant increase in glossiness. An increase in the glossiness values was found when abrasives above 360 were used for the limestone polishing. Moreover, a good correlation between the final glossiness values and the surface roughness (R = 0.92) was found.

From the data presented in Table 3 and Figure 4, a smoothing effect of the black acrylic paint on the surface roughness of aluminum alloy (Al-B-P) and stone (S-B-P) was evident. In the case of glass–paint (G-B-P) and marble–paint (M-B-P), the presence of the paint coating increased the $R_{a}$ and $R_{q}$ parameters. The basic components of acrylic paint are acrylic resins formed through polymerization. In the water-borne acrylic paints the binder component is present in the form of an aqueous suspension of acrylic polymers. Due to its characteristics, acrylic paints form an aesthetic smooth surface on the substrate immediately after drying, which does not change its color even after long periods of time. The advantage of this type of paint is the fastness of drying, covering strength, flexibility and resistance to water, light, and chemical agents. On all substrates with different surface properties and topography, the paint coatings with a similar microstructure were obtained (Figure 3A2–D2). Analyzing the roughness height distribution curves (Figure 4), a symmetrical relationship between the number of events vs. height was observed. For the heights ranging from −3.5 μm to 3.5 μm, it was found that on the surfaces of G-B-P, Al-B-P, S-B-P, and M-B-P, the irregularities occupy 97.5, 96.6, 91.9, and 94.4% of the surfaces, respectively. Nevertheless, there constituted deeper pores on the paint layers as evidenced by the *R_t_* parameter values. During the drying of the paint on the substrate, volatile solvents quickly evaporated, initiating pore formation.

The next stage of the study involved the removal of the graffiti from the investigated sensitive surfaces using the w/o nanoemulsion and the assessment of their surface topography in terms of wettability. Analyzing the amplitude roughness parameters (Table 4) for the two smoothest surfaces, i.e., glass and marble, the microstructure of their surface changed slightly after removing the 500–600 μm thick paint layer. For the glass surface (G-B-Pc) the roughness parameters marginally increased, similarly to the bare glass after cleaning with the eco-cleaner (G-Bc). These minor changes were difficult to attribute unambiguously to the mentioned processes. The flat glass manufactured with the float technique congeals in contact with air and molten tin during the cooling process. The surface of the glass on the tin side is smoother than that on the air side. In practice, it is difficult to distinguish between the two sides of the glass because the roughness on these surfaces is at the nanoscale. In the case of marble after removing the paint coating and cleaning the bare surface (M-B-Pc and M-Bc), the surface was slightly smoothed with a similar distribution of roughness height (Figure 4D).

After removing the graffiti coating from the Al surface (Al-B-Pc), the $R_{a}$ and $R_{q}$ parameters increased while the value of *R_t_* remained unchanged. The roughness height distribution (Figure 4) proved that 97% of the surface was covered with hills (48.1%) and holes (48.9%) ≤ 15 μm. The influence of surface cleaning on its structure was also examined. The purpose of such a surface treatment was to remove impurities and residues, i.e., deposits, dust, fats, oxides, or microorganisms and to increase the surface roughness which in turn, increases the adhesion of the coating and its strength bonding with the substrate [21]. It appeared that after cleaning the aluminum surface (Al-Bc) was slightly rougher than the bare surface (Al-B), which is evidenced by the increase in the $R_{q}$ parameter and the almost 4-fold increase in $R_{t}$ (Table 4).

**Figure 5:**
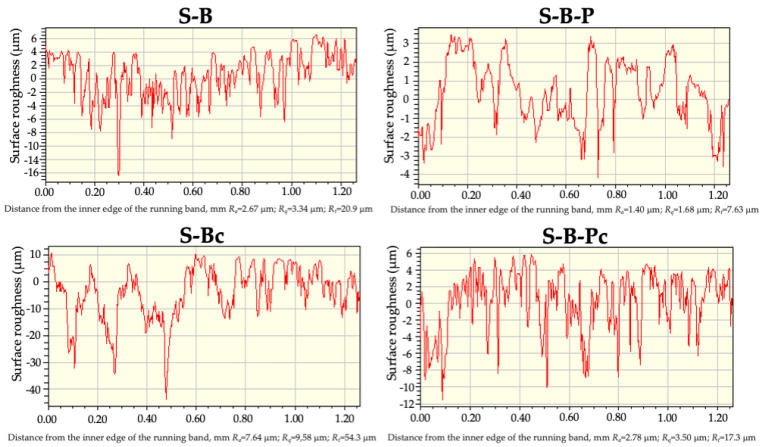
Surface roughness profiles of the running band on the bare stone (S-B), covered with the paint (S-B-P) after its removal and cleaning the bare surface (S-B-Pc). The figure also shows the values of the roughness parameters along the profile curve.

After removing the paint a slightly different distribution of roughness was observed on the stone surface form than on the marble. Almost the entire surface of S-B-Pc had holes (37%) and hills (53%) up to 5 μm. Pores with a depth of up to 15 μm (7.9%) were also exposed, which was clearly visible on the surface roughness profile of the running band (1.2 mm). The process of stone cleaning with the nanoemulsion revealed its porous structure as evidenced by a significant increase in the surface roughness parameters (Table 4 and Figure 5). The roughness height distribution curve on the 1.222 mm^2^ stone surface was flattened (Figure 4), indicating roughness of various dimensions. As follows from the obtained results, the w/o nanoemulsion stabilized by the amino-acid-type surfactants can be used as an ecological remover for graffiti. In addition, it can also serve as an ecological cleaner for various substrates. In the case of stone it should be remembered that these processes uncover pores which can be exposed to the destructive effects of moisture, dirt, and other weather conditions. Therefore, such surface should be protected using an appropriate impregnation process.

#### 2.2.2. Wettability and Surface Free Energy

As mentioned above, the wettability of solids with water provides essential information about the surface properties of materials and this is an important parameter for many industrial processes, e.g., in the metallurgical industry, especially in the corrosion processes of metals, or in the oil industry in the separation of water–oil systems and others [76]. The commonly used parameter for wetting characterization is the contact angle which can range from 0 to 180°. In the physical sense, for the same liquid the value of the contact angle depends on the type of solid and the magnitude of interfacial interactions [77].

**Figure 6:**
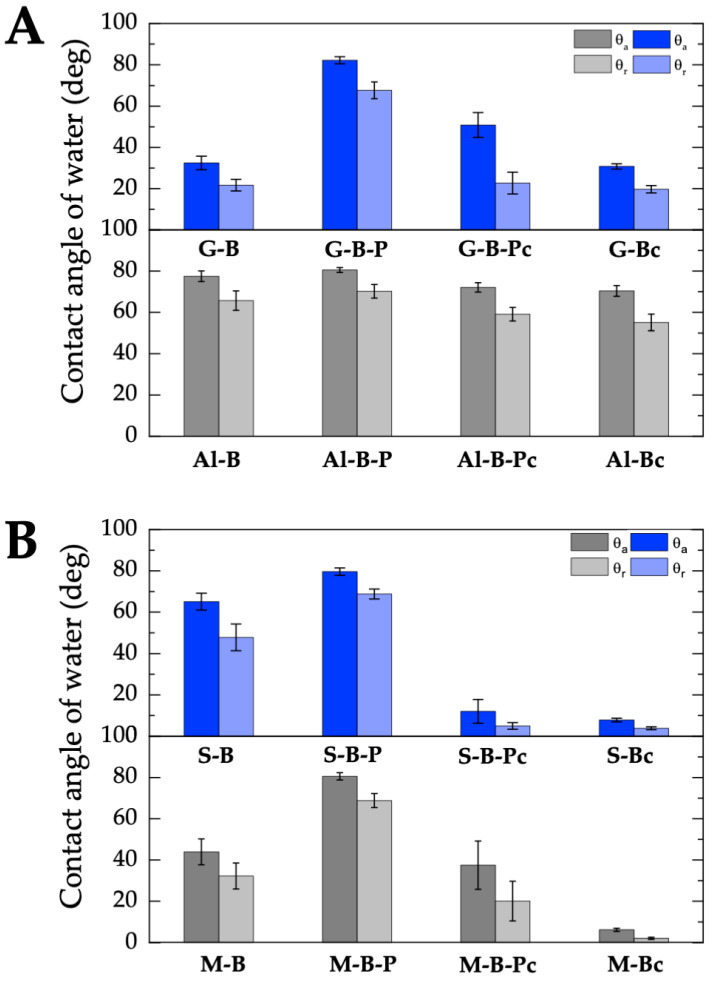
Advancing and receding contact angles of water on the bare sensitive surfaces, covered with paint, after the removal of the paint coating with NE No. 10, and after cleaning the bare surface with NE No. 10. (**A**) Surfaces from the man-made materials. (**B**) Surfaces from the natural materials.

Figure 6 shows the advancing and receding contact angles of water on the bare plates of the man-made materials (glass: G-B and aluminum alloy: Al-B) and on the natural materials (stone: S-B and marble M-B), covered with the black acrylic paint and after removing the paint layers using the ecological remover. Moreover, the contact angles of water on the bare plates after cleaning with the w/o nanoemulsion were measured. Generally, the reproducibility of the measured contact angles was quite good. However, in the case of the bare surfaces of glass, stone, and marble, the contact angle was significantly greater after removal of the paint coating (G-B-Ps, S-B-Ps and M-B-Ps). Obviously significant differences in the structure and chemical composition of natural materials (stone and marble) are found in any measured property. For example, the contact angle values measured on the surface of different specimens originating from the same rock taken from the same quarry are often different [24]. The sensitive bare surfaces were not cleaned to maintain the natural conditions for applying graffiti coatings; hence their surfaces can be energetically heterogeneous. Removal of the graffiti was made by applying the w/o nanoemulsion on the paint coating surface for 10 min and the swelling and dissolving paint was removed using a wet sponge. After the cleaning process, the samples were washed with only demineralized water. It can be assumed that there are areas of different wettability on a visually clean surface which is shown by the measured water contact angles.

**Figure 7:**
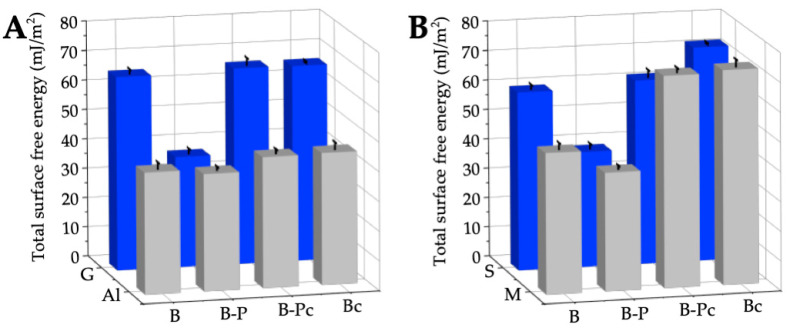
Surface free energy calculated from the CAH approach of bare sensitive surfaces, covered with the paint (B-P), after its removal (B-Pc), and cleaning the bare surfaces with NE No. 10 (Bc). (**A**) The surfaces produced from the man-made materials. (**B**) The surfaces produced from the natural materials.

The type of material, its surface treatment, the surface roughness, and its hydrophobic/hydrophilic properties determine the wettability of the surface with water [24,61]. The smallest water contact angles on the bare plates were obtained on the glass surface (G-B) which is the smoothest of all the tested bare materials. The reproducibility of the advancing and receding contact angle values was very good (vertical bars show standard deviations). The glass surface, the main component of which is SiO_2_, is preferentially wetted by water because of its hydrophilic character due to the presence of hydroxyl groups and siloxane bridges. The hydroxyl (silanol) groups represent strong adsorption sites that interact specifically with water molecules by hydrogen bonding. Water is a polar liquid with a surface tension of 72.8 mN/m at 20 °C whose apolar component is 21.8 mN/m and polar one is 51 mN/m. Hence, the interactions across the water–solid interface are of a dispersive nature and exhibit a polar acid–base character (electron–donor and electron–acceptor). Strong intermolecular interactions occurring at the nanoscale usually lead to strengthening of the interfacial interactions at the solid−liquid interface. At the macroscale, this results in a decrease in the contact angle. On the three studied glass samples: bare glass (G-B), after removing the paint (G-B-Pc) and after cleaning the bare glass (G-Bc), similar water contact angles were obtained. The average advancing contact angle of water was 31.4 ± 1.0°, and the receding one 21.4 ± 1.5°. Analyzing the water contact angles on the smooth glass surface one can observe that the contact angle hysteresis ($H=\theta_{a}-\theta_{r})$ was relatively high (8.2–11.1°). It seems difficult to relate these values of hysteresis with only those of the surface roughness which were only a few nanometers high (Table 4). Starow and Velarde [78] proved that the static advancing contact angle does not depend on the roughness of the solid surface below ∼10–30 nm. Chibowski and Jurak [79] postulated that the hysteresis of the contact angle can result from the presence of the liquid film behind the three-phase solid/liquid drop/gas (vapor) contact line after its withdrawal via the droplet volume reduction as well as the presence of Deriaguin’s disjoining pressure. Obviously, the value of the contact angle hysteresis depends on the intermolecular interactions at the interface, i.e., between the solid surface and liquid. Additional information about the solid surface–liquid interactions can be obtained from the apparent surface free energy calculated from the advancing and receding contact angles and the surface tension of water (Equation (7)) [29,30,31]. Using the measured contact angles (Figure 6) the surface free energies were calculated for the bare sensitive surfaces, those covered with the acrylic paint, after removal of the graffiti coating and after cleaning the bare surfaces with an ecological remover/cleaner as plotted in Figure 7. The obtained total surface free energy values of the glass samples (G-B, G-B-Pc, G-Bc) were similar to those obtained by other authors [80,81,82]. 

Higher values of water contact angles were obtained on the other rougher sensitive surfaces. As already mentioned, the above wettability of metals and their alloys depend on the surface treatment, e.g., adsorption, chemisorption and/or generally interactions of gases present in the environment [25]. Like most metals, aluminum undergoes a natural oxidation process when in contact with atmospheric oxygen, which leads to the formation of a thin layer of aluminum oxide on its surface. The passivation process depends on the smoothness of the aluminum and the created film helps to prevent corrosion. The analysis of XPS results showed that there was about 12.7% aluminum oxide on the surface of the aluminum alloy AMS 4049 [71]. The presence of such a layer should affect the hydrophilic properties of Al and its alloys as well as the surface free energy. On the bare aluminum alloy investigated here, the advancing contact angle of water was 77.5 ± 2.6°, which indicates the hydrophilic nature of the surface [25,62,71,73,74,76]. On the Al-B surface with the average roughness $R_{a}$ = 3.95 ± 0.39 µm, the contact angle hysteresis was 11.8° while the surface free energy determined from Equation (7) was much lower than that of glass amounting to 41.0 ± 2.1 mJ/m^2^ (Figure 6). The smoothing of the roughness of Al-B-P ($R_{a}\;$= 1.36 ± 0.07 µm), caused by the black acrylic paint layer increased its hydrophobic properties ($\theta_{a}\;$= 80.5 ± 1.2°), and slightly decreased the total surface free energy ($\gamma_{s}$ = 39.5 mJ/m^2^) in comparison to the bare surface. 

Pogorzelski et al. [25] carried out a similar study of the wettability of metallic surfaces (Fe, Al, Cu, and a brass alloy) covered with the layers of four paints of different colors (colorless, white, black, and red). They measured the Young’s equilibrium contact angle ($\theta_{Y}$), and the advancing ($\theta_{a}$) and receding ($\theta_{r}$) contact angles of water using the tilting plate method. Based on these experimental data, several surface wettability parameters, such as the contact angle hysteresis (CAH), the film pressure (π), the total surface free energy ($\gamma_{SV}$) calculated from the CAH approach [29,30,31], the work of adhesion ($W_{A}$), and spreading ($W_{S}$), were determined. Pogorzelski et al. [25] found an increase in the hydrophobic properties of the paint-treated metallic surfaces when compared to the bare untreated surfaces. This resulted from the increase in $\theta_{Y}$, $\theta_{a}$, $\theta_{r}$, CAH and a decrease in $\gamma_{SV}$ and $W_{A}$, and a less negative$\;W_{S}$. The increase in the apolar interactions (dispersive component $\gamma_{SV}^{d}$) in relation to the total surface free energy $\gamma_{SV}$ also indicated the surface hydrophobization after its painting. For the untreated metallic surfaces, the $\gamma_{SV}^{d}$/$\gamma_{SV}$ ratio changed in the range (0.74–0.77)$\gamma_{SV}$ while for the paint-treated surfaces it decreased to (0.62–0.69)$\gamma_{SV}$. Moreover, the spatial evolution of the data point distribution in the space of CAH vs. $W_{S}$ allowed the authors to distinguish the processes simultaneously occurring, i.e., micro-roughness smoothing, chemical paint component distribution and mixing at the outermost surface; and found them to be base substratum specific. They associated the surface wettability changes with the compositional changes at the interface but not with the surface roughness. It was because the CAH remained almost the same for both the un-treated and paint-treated metal surfaces. The authors concluded that the CAH methodology using the three measurable quantities, $\theta_{a}$, $\theta_{r}$, and the liquid surface tension, $\gamma_{LV}$, is a useful tool in the studies of such processes as lubrication, liquid coating and thermoflow [25].

In our studies, after removing the black paint (Al-B-Pc) and cleaning the bare aluminum alloy (Al-Bc), the $\theta_{a}$ decreased from 77.5 ± 2.6° to 72.1 ± 2.3°, and 70.2 ± 2.6°, respectively. This shows a minimal increase in the hydrophilicity of these surfaces and a slight increase in their surface free energy (Figure 6). However, no functional relationship was found between the surface roughness (Table 4), wettability (Figure 5) and the surface free energy (Figure 6). 

The advancing water contact angle on such a surface (S-B) was 65.1 ± 4.1° which means that the surface was slightly hydrophilic. It can be assumed that the tiles made of stone were covered with impregnate to protect them against water absorption and dirt penetration. The contact angle decreased to 12 ± 5.8° and 7.8 ± 0.9° after removing the paint (S-B-Pc) and after cleaning the stone (S-Bc), respectively (Figure 6), which exhibits its hydrophilic character, but the roughness height distribution shows that pores of various depths were uncovered (an increase in the roughness parameters) (Table 4 and Figure 4). Generally, according to the Wenzel theory [22], an increase in roughness of the hydrophilic surface (θ < 90°) will enhance its wettability (a decrease in the contact angle), i.e., increase the hydrophilicity. Nevertheless, in the case of the porous materials, the surface roughness can only have a partial effect on the surface wettability, because the imbibition of liquid into the absorbing material is also important [61,83]. On the less porous marble the $\theta_{a}$ of water decreased from 43.9 ± 6.3° (M-B) to 37.5 ± 11.7° (M-B-Pc) and 6.2 ± 0.8° (M-Bc) despite the decrease in the amplitude parameters of surface roughness. 

Regardless of the type of sensitive surfaces (G-B, Al-B, S-B and M-B), the surfaces roughness ($R_{a}$ from 0.37 ± 0.02 nm (G-B) to 3.95 ± 0.39 µm (Al-B)) and their surface free energy ($\gamma_{S}$ from 41.0 ± 2.1 mJ/m^2^ (Al-B) to 65.5 ± 1.4 mJ/m^2^ (G-B)) after being covered with a thick layer of black acrylic paint, the same energetic properties of the solid/paint systems were obtained. The surface free energy of G-B-P, Al-B-P, S-B-P, and M-B-P changed in a narrow range from 37.4 to 39.9 mJ/m^2^. This is consistent with $\gamma_{S}\;$= 39.7 mJ/m^2^ of the acrylic paint determined from the advancing contact angles of two polar (water and formamide), and apolar (diiodomethane) liquids based on the acid–base approach of van Oss et al. [28,84]. The main component of acrylic paint is poly(methyl methacrylate) (PMMA), whose monomer contains −C=O and −O− bonds in the molecule, showing electron–donor interactions. Hence, the PMMA and acrylic paint indicate some weak polar interactions with the predominance of the base ones [84,85,86]. The surface free energy of all the investigated sensitive surfaces–paint layers was relatively low and similar to PMMA [80,87,88,89]. The greatest changes in the surface wettability and surface free energy after removing the paint and cleaning the bare surfaces were found for stone (S-B-Pc and S-Bc) and marble (M-B-Pc and M-Bc), which is related to the hydrophilic nature of natural materials, their porosity and/or the surface treatment (impregnation, or grinding).

#### 2.2.3. Work of Adhesion

**Figure 8:**
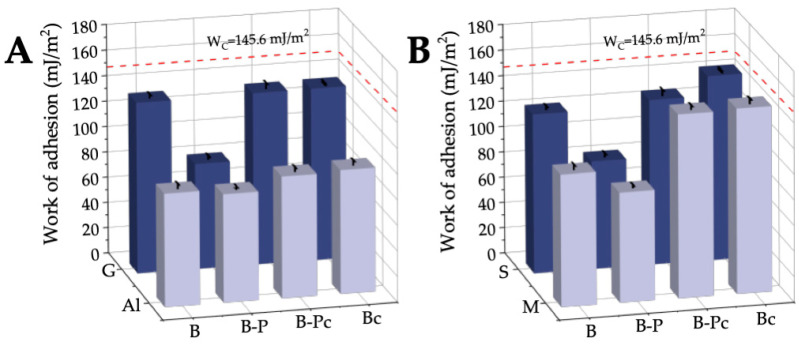
Work of adhesion of water to the bare sensitive surface samples, covered with paint (B-P), after paint removal (B-Pc), and cleaning bare surfaces with NE No. 10 (Bc). (**A**) The surfaces produced from man-made materials. (**B**) The surfaces produced from natural materials.

The changes in the surfaces wettability and surface free energies of the investigated sensitive surfaces can also be well depicted by the changes in the thermodynamic work of adhesion $W_{A}$, which was calculated from the advancing contact angles of water based on the Young–Dupré equation (Equation (5)) (Figure 8). The work of water adhesion is an important parameter because these surfaces are in contact with the water molecules present in the w/o nanoemulsion used to remove the graffiti coating and clean the bare surfaces.

As can be seen in Figure 8, all the values of the work of water adhesion $W_{A}$ were lower than that of the work of water cohesion. Water interacts most strongly with the porous stone surface after removing the paint (S-B-Pc) and after cleaning the bare stone and marble surfaces with the nanoemulsion (S-Bc and M-Bc). For these systems $W_{A}$ was only a little smaller than the work of water cohesion $W_{C}=2\gamma_{L}$ = 2 · 72.8 = 145.6 mJ/m^2^. This indicates the strong hydrophilic character of these surfaces having high surface free energies, greater than those of $\gamma_{S}$ of the hydrophilic bare surfaces (S-B and M-B). In the case of polar glass tiles (G-B, G-B-Pc and G-Bc), the work of water adhesion was lower, amounting to 134.8 ± 0.7 mJ/m^2^. This means that after removing the paint from the smooth glass surface and after cleaning the bare glass, the surface properties of this material did not changed. However, in the case of the other man-made materials, i.e., aluminum, the work of adhesion $W_{A}$ increased slightly, from 88.6 ± 3.2 mJ/m^2^ (Al-B) to 95.2 ± 2.7 mJ/m^2^ (Al-B-Pc) and 96.8 ± 3.3 mJ/m^2^ (Al-Bc). The greatest increase in the work of water adhesion occurred after painting all the sensitive surfaces with the black acrylic paint. The values of $W_{A}$ were from 82.7 ± 2.2 mJ/m^2^ (G-B-P) to 86.2 ± 2.3 mJ/m^2^ (S-B-P) and in the range of the acrylic enamel (84.1 mJ/m^2^) and PMMA (90.3 mJ/m^2^). Hence, the studied layers of graffiti coating on the sensitive surfaces were sufficiently thick to possess properties similar to those of acrylic enamel and PMMA. Therefore, it can be concluded that when removing black acrylic paint from the different sensitive surfaces with w/o nanoemulsion, the kind of substrate should not significantly affect this process. 

### 2.3. “Brush on, Wipe off” Method for Testing Graffiti Removers

The contact angle values of the w/o nanoemulsion are helpful in determining the wettability and adhesion of the graffiti remover to the undesirable paint layers on the sensitive surfaces. A good parameter describing the changes in wettability of paint coatings by nanoemulsion is the work of spreading $W_{S}$, which is expressed by the difference between the work of adhesion, $W_{A}$, and the work of liquid cohesion, $W_{C}$, as expressed by Equation (6). This is a crucial criterion for understanding how the graffiti remover works.

**Figure 9:**
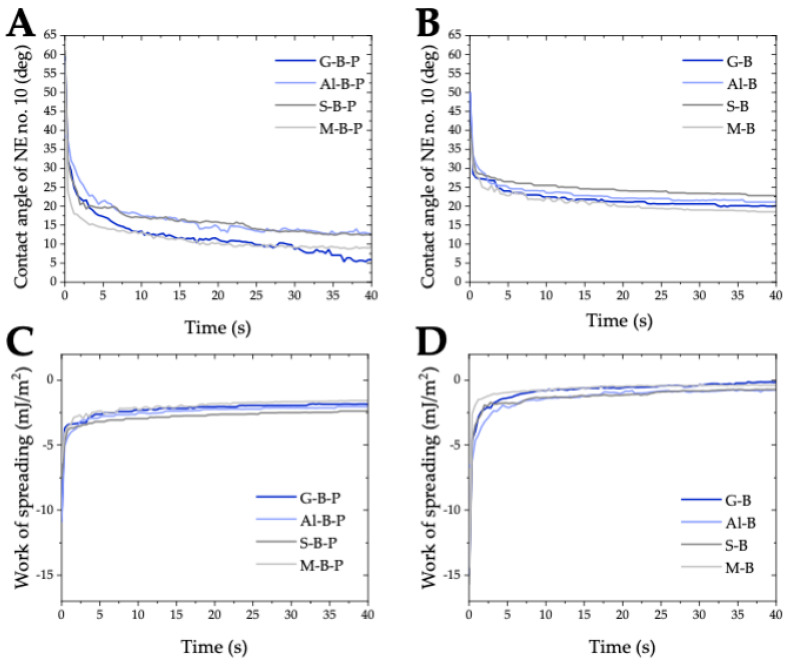
Dynamic contact angles of nanoemulsion No. 10 versus the time contact with the paint covered (**A**) and bare sensitive surfaces (**B**) and its work of spreading on the paint covered (**C**) and bare sensitive surfaces (**D**).

Figure 9A illustrates the changes in the contact angles as a function of time from the moment when the w/o nanoemulsion droplets of nanotechnological detergent, NE No. 10 were deposited on the acrylic black paint layers on different sensitive surfaces (G-B-P, Al-B-P, S-B-P and M-B-P). On all surface–paint systems the contact angles of nanoemulsion decreased sharply during the first 5 s, most extensively on the M-B-P surface (from 58.3 to 14.4°), and least extensively on the Al-B-P surface (from 38.9 to 21.2°). During the next 35 s the reduction in the contact angles was much smaller, and the changes on the surfaces of Al-B-P and S-B-P, and M-B-P and G-B-P were similar. 

Similar experiments were conducted on all the studied bare sensitive surfaces (Figure 9B). Irrespective of the nature of the surface, differences in the surface topography (surface roughness) and free surface energies of the sensitive surfaces, the changes in the nanoemulsion dynamic contact angles were similar. During the first few seconds, the contact angles decreased by about 50%, and then the changes were smaller, in the range from 20 to 25°. Thus, already within 5 s the contact angles of the nanoemulsion droplets on the black acrylic paint layers decreased below the values of the contact angles on bare sensitive surfaces. 

Based on the dynamic contact angles of the nanoemulsion and its surface tension, the work of spreading was calculated (Figure 9C,D). During the first few seconds the work of spreading of the graffiti remover on all tested surfaces, both unpainted and covered with the black paint, was smaller than –5.0 mJ/m^2^. Nevertheless, the values of $W_{S}$ of the w/o nanoemulsion on all the bare surfaces with hydrophilic properties approached zero very quickly, while on more hydrophobic acrylic paint layers they reached the values of about –2.5 mJ/m^2^ within 5 s. This proves the excellent wetting properties of the framed graffiti remover (NE No. 10) sodium cocoyl glycinate stabilized (See Figure 9) on the surfaces characterized by different surface properties.

According to the Shi and Gardner model [90,91], the process of adhesive wetting of porous materials, such as wood, assessed from the measurements of the contact angle, also includes the spreading of the liquid and its penetration into the pores. Hence, contact angle changes as a function of time are observed. The rate at which the liquid penetrates and spreads across the surface of the solid is proportional to the rate at which the contact angle changes. They presented the wetting model of a systems in which the changes in the dynamic contact angles can be quantitatively related to penetration and spreading during the adhesive wetting process by the parameter *K* (the contact angle change rate constant) [90].

(8) $$\theta_{x}=\frac{\theta_{i}\theta_{e}}{\theta_{i}+(\theta_{e}-\theta_{i})exp\left[K\left(\frac{\theta_{e}}{\theta_{e}-\theta_{i}}\right)t\right]}$$

where $\theta_{x}$ is the contact angle at a given moment, $\theta_{i}$ is the initial contact angle, and $\theta_{e}$ is the equibibrium contact angle.

The constant *K* value allows to assess how fast the liquid spreads over the surface and penetrates into the solid pores. If the *K*-value is higher than zero, then the contact angle will approach its equilibrium value more quickly, and the liquid will penetrate and spread across the surface more quickly as well. There will be no penetration or spreading of the liquid over the surface when *K* is equal to zero and the equilibrium contact angle is equal to the initial contact angle. On the other hand, when *K* approaches high values, it is safe to assume that the liquid quickly wets the surface of the solid (this means that the contact angle is 0) [90]. In addition to evaluating the spreading of adhesives on the surface of the wood, this model can also be used to evaluate the efficacy of a given graffiti remover and to determine whether or not it is suitable for removing particular paint coatings.

In our study, the droplets of the nanotechnological detergent (NE No. 10) were spread on the bare sensitive surfaces but did not penetrate into the pores of natural materials as shown by the achievement of a stable equilibrium contact angle after 5 s. This confirms that the detergent does not destroy the natural materials. However, the interactions of this ecological preparation with the porous coats of graffiti paint additionally results in the penetration of the nanoemulsion into the porous structure of the paint (see Figure S1).

The majority of the previous studies on the thermodynamics of the interaction between the apolar paint coats and nanostructured liquid showed the usefulness of the temporary or equilibrium contact angles [49,92]. This is due to the fact that these contact angles are easier to measure. Therefore, the process of liquid entering and spreading was not taken into consideration. However, the capacity to penetrate in addition to the graffiti remover’s exceptional spreading is a vital factor for assessing the capability and efficiency of removing undesired coatings from sensitive surfaces. This property was determined by the graffiti remover spreading ability. Hence, having knowledge of the adhesive wetting process of the nanostructured paint surface fluid, including all the data regarding the formation of drops, their spreading on the surface, and ability to penetrate into the coating structure, allows to develop a new generation of graffiti removal agents that are based on nanotechnological detergents. They could be designed to remove paint safely without causing any surface damage.

## 3. Materials and Methods

### 3.1. Materials

All the studied amino-acid-type surfactants were obtained as commercial reagents from Clariant Produkte (Frankfurt am Main. Germany) (for the abbreviation, see Table 1). Ethyl lactate (Purasolv EL, Corbion) was purchased from Envolab (Długołomice, Poland). Our research team manufactured the used cooking oil PEG-8 ester solvent [41]. The surface samples were purchased from DellArte Group Sp. z o.o. (Robakowo, Poland) (for the abbreviation, see Figure 2). The spray paint Montana Black was purchased from Montana Cans™ (MONTANA-CANS. Heidelberg. Germany). The organic solvents, acids, and hydroxides were of analytical grade and were obtained from Avantor™ and delivered by VWR™ (Gliwice, Poland).

### 3.2. Fabrication of NSFs

The formulation was prepared using the selected amino acid surfactants (AAS structures 1–4, Table 1) at different concentrations. The continuous phase (78.5%) was composed of biosolvents, i.e., the cooking Oil-PEG-8 ester (38.5%) and ethyl lactate (40%). The dispersed phase included water (14%), and different types of amino acid (0.05; 0.075; 0.1 mol/dm^3^) surfactants were used. Twelve sets of 250 mL pre-emulsions (see Table S1) were prepared by normal-pressure mechanical homogenization at 700 rpm for 5 min with the rotor-stator stirrer (IKA Works GmbH & Co. KG, Staufen, Germany) equipped with the 4-bladed propeller stirrer at 25 °C. The resulting w/o nanoemulsions were prepared according to the high-energy method, i.e., high-pressure homogenization. The abovementioned pre-emulsions were passed through the air-operated laboratory-scale high-pressure LV1 homogenizer (Microfluidics, Newton, MA, USA). Its basic construction includes the orifice-type valve (1.0 mm diameter). The homogenized fluid escapes the device head after impacting a cone-shaped metallic piece. The maximum shear rate generated by LV1 at 150 MPa pressure was 9.23⋅106 s^–1^. The inlet temperature of the w/o nanoemulsion (100 mL sample volume) was maintained at 25 ± 2 °C, and the homogenization pressure was set to either 100 MPa or 150 MPa. Each of the prepared emulsions was passed through the head of the LV1 microfluidizer in five separate cycles (1 cycle: inlet, high shear rate, and outlet of the fluid).

### 3.3. Physicochemical Characterization Measurements and RSM Optimization

#### 3.3.1. Surface Tension Measurements

The surface tension of NSF at 20 ± 1 °C was determined using the Theta Lite optical tensiometer based on the hanging drop shape analysis and the Young–Laplace equation.

#### 3.3.2. Contact Angle Measurements

The advancing ($\theta_{a}$) and receding ($\theta_{r}$) contact angles of water on the studied sensitive surfaces were measured using a GBX contact angle meter (France) equipped with a digital camera and chamber enabling the adjustment of temperature and relative humidity. The contact angles were determined based on the deposited drop shape analysis using the WinDrop software. The measurements of the advancing contact angle were performed using the water droplet of 6 μL, which was settled on the surface of the plate with a microsyringe. The receding angle was measured after removing 2 μL of water from the droplet on the surface. The measurements were made at the temperature of 20 ± 1 °C and 50% humidity. The figures show the mean values of contact angles on both sides from 10 to 15 water droplets. For each series of measurements, the standard deviation from the mean value was determined.

#### 3.3.3. Surface Free Energy Determination

The total surface free energy ($\gamma_{S})\;$ of the studied solid support was determined from the contact angle hysteresis (CAH) model proposed by Chibowski [29,30,31] based on the measured advancing and receding contact angles of water and its surface tension ($\gamma_{LV}$) at 20 °C.

#### 3.3.4. Surface Topography

The topography of the studied surfaces was estimated by the optical profilometer (Contour GT-K1, Bruker, Germany) using the VXI measurement mode or the extended vertical scanning interferometry (VSI).

#### 3.3.5. Dynamic Light Scattering (DLS)

The droplets size was investigated using the dynamic light scattering (DLS) by means of the Malvern Zetasizer Nano ZS (Malvern Instruments, UK). Five measurements were performed at room temperature (20 ± 5 °C) to obtain a single average result. Each sample was evaluated in its undiluted form.

#### 3.3.6. Turbidimetric Measurements

After the high-pressure homogenization treatment, the stability of the nanoemulsion was examined in the Turbiscan LabExpert (Formulaction, Smart Scientific Analysis, Toulouse, France) for 3 months at 20 °C. The w/o nanoemulsion sample in a glass vial was placed in the thermostatic chamber. The electroluminescence diode emitted a collimated light beam (λ = 880 nm) passing through the sample. The transmission detector recorded the light passing through the sample at the angle of 0° in the incident light direction. The other diode acted as a backscattering detector recording the light scattered at the angle of 135°. In addition, the Turbiscan stability index (TSI) values were computed using the Turbiscan Easy Soft (Formulaction, Smart Scientific Analysis, Toulouse, France).

#### 3.3.7. RSM Optimization

The response surface methodology (RSM) was utilized to determine the optimal parameters for fabrication of w/o nanoemulsion-based graffiti removers by the high-pressure homogenization process. The Design Expert software (ver. 13.0.12.0, State-Ease, Minneapolis, MI, USA) was employed to study the randomized quadratic D-optimal model in the coordinate exchange mode through the response surface exploration [41,42]. To find out the best combination of process parameters, a custom-built (3–4)^3^ factorial D-optimal design (shown in ESI Table S3) was applied. In this study the independent variables, with their corresponding levels, were as follows: the concentration of AAS (A) at 3 levels ((–1) 0.050 mol/dm^3^; (0) 0.075 mol/dm^3^; (+1) 0.100 mol/dm^3^); the homogenization pressure (B) at 3 levels ((–1) 1 atm; (0) 1000 atm; (+1) 1500 atm) and the type of AASs used in the w/o nanoemulsion at 4 levels ((–2) SLG; (–1) SCCG; (0) SMCT; (+1) SCG). In the presented investigation 12 candidate experiments (see Table 2) served as a library to form the 32-run D-optimal experimental matrix. The RSM study was used to determine the existing relationship between the independent input variables and the response factors of the optimized homogenization process. The crucial physical characteristics of the fabricated nanoemulsions, i.e., particle diameter, PDI, and emulsion stability (TSI after 0, 7 and 30 days), were used as the response factors: (Y_1_), (Y_2_), (Y_3_), (Y_4_), and (Y_5_). The existing correlation between the independent and dependent variables is defined by the second-order polynomial formula derived from the D-optimal design, (see Equation (S1)) [42]. The analysis of variance (ANOVA), followed by the resulting statistical metrics (*p*- and F-values, and R^2^), allowed for evaluation of the selected optimization model and fitting of the predicted and actual experimental data. The optimal region of parameters for preparing AAS-based w/o nanoemulsions was determined using the 3D response surfaces modeled from the Y_1_–Y_5_ polynomial equations (Equations (S2)–(S6)).

#### 3.3.8. Optical Microscopy Analysis

The surface and microstructure of the sensitive surfaces were examined using an Eclipse E600POL polarizing optical microscope (Nikon, Tokyo, Japan) at magnifications of 40× and 100×.

#### 3.3.9. Laboratory “Brush on, Wipe off” Detergent Tests

A laboratory “brush on, wipe off” detergent assessment was made with the proposed optimal w/o nanoemulsion graffiti remover formulation. The tests were performed on distressed black paint under laboratory conditions (20 ± 5 °C, 40 ± 5% RH, 30 days) applied to reference surfaces, i.e., glass, aluminum, marble, and stone. Small droplets of the selected formulation (4–5 mL) were overlaid on the surface of the paint coating (coating layer thickness: 500–600 µm), and interaction with the painted layer was allowed for 10 min. Afterward, the w/o nanoemulsion with a swelling and dissolving paint coating was gently removed by performing mechanical abrasion with a humid sponge. After the cleaning process was completed, the sample surfaces were washed with fully demineralized water. The cleaning procedure was repeated for reference on sensitive surfaces that were not covered with paint.

## 4. Conclusions

These investigations were aimed at demonstrating some key aspects of the interactions between graffiti paint coatings applied to various sensitive surfaces (glass tiles, aluminum, stone, and marble) and the w/o nanoemulsion stabilized with AAS. The nature of sensitive surfaces and graffiti coatings that must be removed and the effect of the nanoemulsion on bare and black acrylic paint-coated plates were the main issues considered in this present contribution. Optimization through the RSM was performed and allowed to formulate the most effective eco-graffiti remover identified as NE No. 10. These experiments proved that the type of amino acid in the AAS molecule (with the simplest structure, i.e., glycine) is crucial for the stabilization of nanoemulsion systems. Based on the wettability of paint coatings with a graffiti remover, it seems that the action of this type of agent was due to the intermolecular interactions of the w/o nanoemulsion and the apolar surface of the paint. Additionally, the emulsion spreading and its penetration rate into the pores formed during the graffiti coating drying played the most significant role.

However, regardless of the size of the surface roughness, after coating them with a relatively thick layer of black acrylic paint, the obtained solid–paint systems possessed practically the same surface properties and topography. Depending on the kind of substrate and its polarity, the surface free energies after coating with the paint decreased by about 4% (Al-B-P) to 43% (G-B-P), thus reaching the value typical of acrylic paints (37.4–39.9 mJ/m^2^). It seems that the removal of graffiti coatings from sensitive surfaces with the use of a nanotechnological detergent initially was more dependent on the energetic properties and microporous structure of the paint layer than on the properties of substrates on which the layer was deposited. Moreover, the graffiti coating eco-remover proposed by us ensures that the original substrate is not affected during the removal of the undesirable coating. It is believed that this work can be an opportunity for the further study and development of “brush on, wipe off” cleaning methods. Using nanostructured fluids and applying knowledge about the properties of the surfaces could enable one to develop efficient detergents that would remove easily the unwanted contaminants from different surfaces in a fully controlled way.

## Figures and Tables

**Table 1:** Structures and abbreviations for the amino-acid-type surfactants (AAS) and biosolvents.

No.	Structure	INCI	Abb.	M_w_ (M)	$\gamma_{LV}$^1^ (mN/m)	HLB_m_^2^
Amino-acid-type surfactants (AAS)						
1	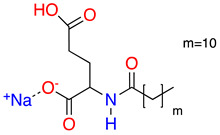	Sodium Lauroyl Glutamate	SLG	356	35.7	9.7
2	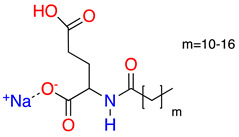	Sodium Cocoyl Glutamate	SCCG	359	29.9	8.3
3	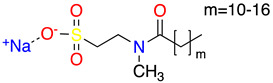	Sodium Methyl Cocoyl Taurate	SMCT	363	35.3	6.1
4	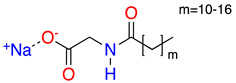	Sodium Cocoyl Glycinate	SCG	273	26.4	7.5
Solvents						
1	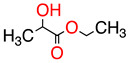	Ethyl lactate	EL	118.1	28.7	8.3
2	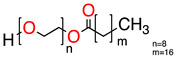 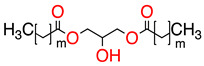	UCO-Oil-PEG 8	UCO-Oil-PEG 8	636.9 625.0	32.6	3.5 0.9

^1^ Measured by the sessile drop methods (see 2.4.1) ^2^ Calculated according to the universal McGowan scale [46].

**Table 2:** Physicochemical characterization of the products after HPH at P = 150 MPa and after five cycles of homogenization.

Type of AAS:	SLG			SCCG			SCMT			SCG		
NE No.	1	2	3	4	5	6	7	8	9	10	11	12
Concentration (mol/dm^3^)	0.05	0.075	0.1	0.05	0.075	0.1	0.05	0.075	0.1	0.05	0.075	0.1
0 days storage												
D_H_ (nm)	749	1042	1424	1328	1963	2247	**435 ^1^**	776	817	**186**	**478**	761
D_H_ S.D. ± (nm)	143	190	253	297	460	695	96	150	152	36	113	198
PDI	0.036	0.033	0.032	0.050	0.055	0.096	0.049	0.037	0.035	0.037	0.056	0.068
TSI	1.01	1.57	2.40	1.74	1.89	2.27	0.86	1.53	1.56	0.06	0.09	0.11
7 days storage												
D_H_ (nm)	779	1137	1524	- ^2^	-	-	461	864	906	192	497	799
D_H_ S.D. ± (nm)	144	209	281	-	-	-	102	167	175	38	117	208
PDI	0.034	0.034	0.034	-	-	-	0.049	0.037	0.037	0.038	0.055	0.068
TSI	1.67	3.05	5.04	31.32	34.02	31.78	1.17	2.74	2.84	0.07	0.10	0.20
1 month storage												
D_H_ (nm)	812	1194	1618	-	-	-	484	916	961	200	514	833
D_H_ S.D. ± (nm)	146	215	292	-	-	-	104	178	187	39	122	219
PDI	0.032	0.032	0.033	-	-	-	0.046	0.038	0.038	0.038	0.057	0.069
TSI	2.12	4.69	8.42	-	-	-	1.35	3.39	3.80	0.10	0.15	0.78
3 months storage												
D_H_ (nm)	843	253	1681	-	-	-	508	971	1018	205	533	868
D_H_ S.D. ± (nm)	147	221	304	-	-	-	106	91	209	42	127	238
PDI	0.030	0.031	0.033	-	-	-	0.044	0.039	0.042	0.042	0.057	0.075
TSI	2.35	5.44	10.02	-	-	-	1.51	4.17	4.76	0.15	0.22	1.64

^1^ Bold values represent the products obtained at the nanoscale. ^2^ No data collected due to the lack of time-dependent stability.

**Table 3:** Candidates proposed by the D-optimal model optimization for the best AAS-based w/o nanoemulsion graffiti eco-remover based on the desirability function and the correlation of the observed and predicted values.

No.	NE No.	Type of AAS	Diameter (nm)		PDI		TSI (0 Days)		TSI (7 Days)		TSI (30 Days)		Desirability
			P ^3^	A ^4^	P	A	P	A	P	A	P	A	
1	10 ^1^	SLG	207	186	0.042	0.037	0.06	0.06	1.66	0.07	0.34	0.10	0.964
2	11 ^2^	SLG	380	478	0.043	0.057	0.20	0.09	1.56	0.10	5.53	0.15	0.950

^1^ As presented in Table 2, obtained at the 0.05 mol/dm^3^ AAS concentration under the HPH pressure of 150 MPa. ^2^ As presented in Table 2, obtained at the 0.75 mol/dm^3^ AAS concentration under the HPH pressure of 150 MPa. ^3^ Values predicted by the D-optimal model. ^4^ Actual experimental values.

**Table 4:** Roughness parameters of the sensitive bare surfaces: glass (G-B), aluminum alloy (Al-B), stone (S-B), marble (M-B), painted (B-P), after removal of paint coating (B-Pc) and cleaning the bare surface with nanoemulsion (Bc).

Sample	$R_{a}$	$R_{q}$	$R_{t}$
	(nm)		
G-B	0.37 ± 0.02	0.62 ± 0.07	28.9 ± 17.1
G-B-P	1.16 ± 0.02 μm	1.48 ± 0.01 μm	19.2 ± 4.5 μm
G-B-Pc	0.74 ± 0.07	1.60 ± 0.39	99.5 ± 52.2
G-Bc	0.73 ± 1.63	1.34 ± 0.29	47.6 ± 16.7
	(μm)		
Al-B	3.95 ± 0.39	5.01 ± 0.56	46.0 ± 4.1
Al-B-P	1.36 ± 0.07	1.69 ± 0.04	14.3 ± 0.9
Al-B-Pc	5.42 ± 0.24	6.72 ± 0.22	44.4 ± 4.5
Al-Bc	3.97 ± 0.24	5.69 ± 0.38	179.0 ± 19.5
S-B	3.78 ± 1.40	5.08 ± 1.90	49.2 ± 9.7
S-B-P	1.62 ± 0.04	2.05 ± 0.06	17.1 ± 0.7
S-B-Pc	2.72 ± 0.30	3.50 ± 0.38	51.8 ± 8.3
S-Bc	6.55 ± 0.82	8.56 ± 1.04	72.4 ± 1.4
M-B	1.12 ± 0.20	1.86 ± 0.13	32.8 ± 12.8
M-B-P	1.50 ± 0.16	1.92 ± 0.22	20.8 ± 3.70
M-B-Pc	0.69 ± 0.05	1.38 ± 0.26	23.2 ± 5.50
M-Bc	0.81 ± 0.05	1.27 ± 0.35	20.1 ± 17.2

## Data Availability

Data will be available upon request.

## Supplementary Materials

The following supporting information can be downloaded at https://www.mdpi.com/article/10.3390/molecules28041986/s1. Figure S1. Image of the painted sensitive surfaces with black paint enlarged by 100×. Table S1. Physicochemical characteristics of the product after atmospheric homogenization (P = 0.1 MPa. 700 RPM). Table S2. Physicochemical characterization of the products after HPH at P = 100 MPa and after five cycles of homogenization. Equation (S1). The second-order polynomial function derived from the D-optimal design model. Table S3. The custom built (3–4)^3^ factorial D-optimal design with corresponding variables and their levels. Table S4. A quadratic D-optimal randomized design experimental matrix of three independent variables with their corresponding values and analyzed response factors, Y_1_–Y_5_: particle diameter, PDI, TSI after 0 days, TSI after 7 days, and TSI after 30 days, respectively. Text S1. Description of the ANOVA evaluation of the D-optimal model fitting of the experimental results, followed by analysis of the relationship between the independent and dependent variables. Equations (S2)–(S6). Polynomial regression equations that emerged from the ANOVA analysis of the D-optimal model for particular response factors. Table S5. ANOVA results for the D-optimal randomized design quadratic model for dependent variables of the graffiti remover w/o nanoemulsion formulations. Figure S2. A graphical representation of the randomized quadratic D-optimal design response surfaces for the dependent variables Y_1_ = diameter, Y_2_ = PDI, Y_3_ = TSI (0 days), Y_4_ = TSI (7 days), and Y_5_ (TSI 30 days) vs. the independent variables (concentration of AAS (A), HPH pressure (B) as a function of the AAS type used: SLG). Figure S3. A graphical representation of the randomized quadratic D-optimal design response surfaces for the dependent variables Y_1_ = diameter, Y_2_ = PDI, Y_3_ = TSI (0 days), Y_4_ = TSI (7 days), and Y_5_ (TSI 30 days) vs. the independent variables (concentration of AAS (A), HPH pressure (B) as a function of the AAS type used: SCCG). Figure S4. A graphical representation of the randomized quadratic D-optimal design response surfaces for the dependent variables Y_1_ = diameter, Y_2_ = PDI, Y_3_ = TSI (0 days), Y_4_ = TSI (7 days), and Y_5_ (TSI 30 days) vs. the independent variables (concentration of AAS (A), HPH pressure (B) as a function of the AAS type used: SCMT). Figure S5. A graphical representation of the randomized quadratic D-optimal design response surfaces for the dependent variables Y_1_ = diameter, Y_2_ = PDI, Y_3_ = TSI (0 days), Y_4_ = TSI (7 days), and Y_5_ (TSI 30 days) vs. the independent variables (concentration of AAS (A), HPH pressure (B) as a function of the AAS type used: SCG). [93,94,95].

## Sample Availability

All samples are available from the authors.
