# Supplementary material for: Surface Properties of Graffiti Coatings on Sensitive Surfaces Concerning Their Removal with Formulations Based on the Amino-Acid-Type Surfactants
Source: Molecules. 2023 Feb 20;28(4):1986. doi: 10.3390/molecules28041986 (PMC9958821; doi:10.3390/molecules28041986)
Supplement: Supplementary file 1 [file molecules-28-01986-s001.zip › molecules-2218290-supplementary.pdf]

SUPPLEMENTARY MATERIAL

Article

Surface properties of graffiti coatings on sensitive surfaces concerning their removal with formulations based on the amino-acid type surfactants

Marcin Bartman [1]. Sebastian Balicki [1]. Lucyna Hołysz [2] and Kazimiera A. Wilk [1, *]

[1] Department of Engineering and Technology of Chemical Processes. Wrocław University of Science and Technology. Wybrzeże Wyspiańskiego 27. 50-370 Wrocław. Poland

[2] Institute of Chemical Sciences. Faculty of Chemistry. Maria Curie-Skłodowska University. Plac M. Curie-Skłodowska 3. 20-031 Lublin. Poland

* Correspondence: kazimiera.wilk@pwr.edu.pl; Tel. +48 71 320 2828

Table of Contents

Figure S1. Image of the painted with black paint of sensitive surfaces with a enlargement of 100x

Table S1. Physicochemical characteristic of the product after atmospheric homogenization (P = 0.1 MPa. 700 RPM).

Table S2. Physicochemical characterization of the products after HPH at P = 100 MPa and after five cycles of homogenization.

Equation S1. The second-order polynomial function derived from the **D-**optimal design model.

Table S3. The custom built $(3–4)^3$ factorial D-optimal design, with corresponding variables and their levels.

Table S4. A quadratic D-Optimal randomized design experimental matrix of three independent variables with their corresponding values and analyzed response factors $Y_1 – Y_5$: particle diameter, PDI, TSI after 0 days, TSI after 7 days, and TSI after 30 days respectively.

Text S1. Description of the ANOVA evaluation of the D-optimal model fitting of the experimental results, followed by analysis of the relationship between independent and dependent variables.

Equation S2 – S6. polynomial regression equations that emerged from the ANOVA analysis of D-optimal model for particular response factors.

Table S5. ANOVA results for D-optimal randomized design quadratic model for dependent variables of graffiti remover w/o nanoemulsion formulations.

Figure S2. A graphical representation of the randomized quadratic *D*-optimal design response surfaces for the dependent variables Y_1 = diameter, Y_2 = PDI, Y_3 = TSI (0 days), Y_4 = TSI (7 days), and Y_5 (TSI 30 days) vs. independent variables (concentration of AAS (A), HPH pressure (B) as a function of AAS type used: SLG).

Figure S3. A graphical representation of the randomized quadratic *D*-optimal design response surfaces for the dependent variables Y_1 = diameter, Y_2 = PDI, Y_3 = TSI (0 days), Y_4 = TSI (7 days), and Y_5 (TSI 30 days) vs. independent variables (concentration of AAS (A), HPH pressure (B) as a function of AAS type used: SCCG).

Figure S4. A graphical representation of the randomized quadratic *D*-optimal design response surfaces for the dependent variables Y_1 = diameter, Y_2 = PDI, Y_3 = TSI (0 days), Y_4 = TSI (7 days), and Y_5 (TSI 30 days) vs. independent variables (concentration of AAS (A), HPH pressure (B) as a function of AAS type used: SCMT).

Figure S5. A graphical representation of the randomized quadratic *D*-optimal design response surfaces for the dependent variables Y_1 = diameter, Y_2 = PDI, Y_3 = TSI (0 days), Y_4 = TSI (7 days), and Y_5 (TSI 30 days) vs. independent variables (concentration of AAS (A), HPH pressure (B) as a function of AAS type used: SCG).

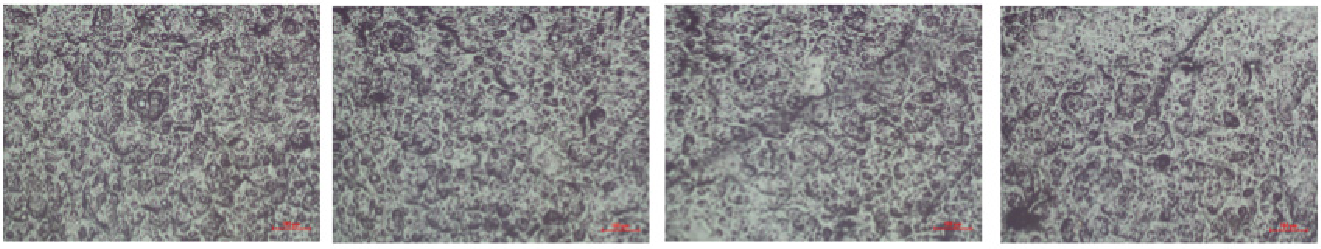

Surface: Glass (G-B-P) | Surface: Aluminium (Al-B-P) | Surface: Stone (S-B-P) | Surface: Marble (M-B-P)

Figure S1. Image of the painted with black paint of sensitive surfaces with a enlargement of 100x

Table S1. Physicochemical characteristic of the product after atmospheric homogenization (P = 0.1 MPa. 700 RPM).

	SLG			SCCG			SCMT			SCG		
Pre-emulsion no.	1	2	3	4	5	6	7	8	9	10	11	12
Concentration (mol/dm^3)	0.05	0.075	0.1	0.05	0.075	0.1	0.05	0.075	0.1	0.05	0.075	0.1
0 days storage												
D_H (μm)	4.768	6.537	8.322	5.213	9.372	11.013	3.523	4.824	5.152	1.162	3.389	5.151
D_H S.D. ± (μm)	0.942	1.046	1.288	0.877	2.573	4.124	0.772	0.887	0.976	0.232	0.693	0.886
PDI	0.039	0.026	0.024	0.028	0.075	0.140	0.048	0.034	0.036	0.040	0.042	0.030
TSI (60 min storaged)	1.41	2.09	3.64	2.74	3.47	5.13	1.35	2.43	2.52	0.11	0.13	0.16
TSI 7 days storage	2.54	5.17	8.58	25.45	45.27	79.64	3.19	5.09	6.68	2.70	2.83	3.35
TSI 1 months storage	5.02	7.06	10.69	x	x	x	6.71	8.46	10.54	4.91	5.93	6.90
TSI 3 months storage	8.88	12.56	18.61	x	x	x	9.66	12.19	15.28	6.14	7.29	8.70

Table S2. Physicochemical characterization of the products after HPH at P = 100 MPa and after five cycles of homogenization.

	SLG			SCCG			SCMT			SCG		
NE no.	1	2	3	4	5	6	7	8	9	10	11	12
Concentration (mol/dm^3)	0.05	0.075	0.1	0.05	0.075	0.1	0.05	0.075	0.1	0.05	0.075	0.1
0 days storage												
D_H (nm)	2537	3580	4696	2710	4790	5763	1696	2401	2473	746	1984	2720
D_H S.D. ± (nm)	494	648	803	631	1312	2103	297	479	498	109	318	462
PDI	0.038	0.033	0.029	0.054	0.075	0.133	0.031	0.040	0.041	0.021	0.026	0.029
TSI (60 min storaged)	1.51	2.26	3.35	1.82	2.26	3.31	1.02	1.95	2.21	0.09	0.11	0.14
TSI 7 days storage	2.73	4.75	8.05	25.41	36.15	57.99	2.40	4.97	5.84	0.84	1.10	1.56
TSI 1 months storage	6.54	13.29	26.55	x	x	x	6.01	15.40	18.68	2.36	3.19	4.68
TSI 3 months storage	7.20	15.15	31.60	x	x	x	6.37	16.93	20.92	3.07	4.28	6.55

The existing relationship between response factors and independent variables is represented by the second-order polynomial function derived from the D-optimal design model:

$$Y_1, Y_2, Y_3, Y_4 \text{ or } Y_5 = \beta_0 + \beta_1 A + \beta_2 B + \beta_3 C + \beta_{1,2} AB + \beta_{1,3} AC + \beta_{2,3} BC + \beta_{1,1} A^2 + \beta_{2,2} B^2 + \beta_{3,3} C^2 \quad \textbf{(Eq. S1):}$$

where $Y_1 - Y_5$ are the dependent variables; A, B and C are independent variables; β_0 is an intercept term; β_1, β_2 and β_3 are the linear coefficients; $\beta_{1,2}$, $\beta_{1,3}$ and $\beta_{2,3}$ are the interaction coefficients; and $\beta_{1,1}$, $\beta_{2,2}$ and $\beta_{3,3}$ are the quadratic coefficients.

Table S3. The custom built (3–4)^3 factorial D-optimal design, with corresponding variables and their levels.

Independent variables	Level			
	**-2**	**– 1**	**0**	**+ 1**
(A) = concentration of amino acid based surfactant (mol/dm^3)	-	0.050	0.075	0.100
(B) = homogenization pressure (atm)	-	1	1000	1500
(C) = type of surfactant used	SLG	SCCG	SCMT	SCG
**Dependent variables**	**Goal**			
Y_1 = particle diameter (μm)	Minimalize			
Y_2 = PDI	Minimalize			
Y_3 = TSI (0 days)	Minimalize			
Y_4 = TSI (7 days)	Minimalize			
Y_5 = TSI (30 days)	Minimalize			

Table S4. A quadratic D-Optimal randomized design experimental matrix of three independent variables with their corresponding values and analyzed response factors Y_1 – Y_5: particle diameter, PDI, TSI after 0 days, TSI after 7 days, and TSI after 30 days respectively.

Run	A: Concentration of AAS [mol/dm^3]	B: Pressure [atm]	C: AAS type	Y_1: diameter [μm]	Y_2: PDI	Y_3: TSI (0 days)	Y_4: TSI (7 days)	Y_5: TSI (30 days)
1	0.05	100	SCCG	2.71	0.0542151	1.81525	25.4135	25.4135
2	0.1	0.1	SCG	5.151	0.0295859	0.16	3.35256	6.9033
3	0.075	150	SCG	0.478	0.0558858	0.090688	0.0997568	0.149635
4	0.1	150	SMCT	0.817	0.0346218	1.56	2.8392	3.80453
5	0.05	100	SCG	0.746	0.0213489	0.08558	0.842107	2.3579
6	0.1	0.1	SCCG	11.013	0.140225	5.13	79.637	79.637
7	0.05	150	SMCT	0.435	0.0487039	0.856417	1.17329	1.34929
8	0.075	0.1	SCG	5.151	0.0295859	0.16	3.35256	6.9033
9	0.1	150	SCCG	2.247	0.0956673	2.27014	31.7819	31.7819
10	0.075	100	SMCT	2.401	0.0398003	1.94752	4.96618	15.3952
11	0.075	100	SLG	3.58	0.032763	2.26	4.746	13.2888
12	0.075	100	SCCG	4.79	0.0750234	2.25916	36.1466	36.1466
13	0.075	150	SCG	0.478	0.0558858	0.090688	0.0997568	0.149635
14	0.05	150	SCCG	1.328	0.0500169	1.74	31.32	31.32
15	0.1	100	SLG	4.696	0.0292398	3.35235	8.04565	26.5507
16	0.1	100	SCG	2.72	0.02885	0.1352	1.55886	4.67657
17	0.1	150	SLG	1.424	0.0315661	2.4	5.04	8.4168
18	0.05	0.1	SLG	4.768	0.0390328	1.41	2.54	5.02
19	0.05	150	SLG	0.749	0.0364509	1.01	1.6665	2.11646
20	0.1	0.1	SLG	8.322	0.0239539	3.64386	8.5774	10.6937
21	0.05	0.1	SCCG	5.213	0.0283024	2.73571	25.4486	25.4486
22	0.1	0.1	SMCT	5.152	0.0358879	2.52335	6.68001	10.539
23	0.1	150	SCCG	2.247	0.0956673	2.27014	31.7819	31.7819
24	0.05	0.1	SCG	5.151	0.0295859	0.16	3.35256	6.9033
25	0.05	150	SLG	0.749	0.0364509	1.01	1.6665	2.11646
26	0.075	0.1	SMCT	4.824	0.033809	2.43	5.09155	8.46323
27	0.1	150	SMCT	0.817	0.0346218	1.56	2.8392	3.80453
28	0.05	0.1	SMCT	3.523	0.0480186	1.35	3.19415	6.71116
29	0.1	0.1	SCCG	11.013	0.140225	5.13	79.637	79.637
30	0.075	0.1	SLG	6.537	0.0256039	2.09	5.17	7.06
31	0.05	150	SCG	0.186	0.037461	0.0620756	0.0651794	0.0977691
32	0.05	150	SCG	0.186	0.037461	0.0620756	0.0651794	0.0977691

Text S1.

The main goal of the optimization was to obtain a w/o nanoemulsion with an average particle diameter on the nanoscale (less than 500 nm, then better) and as low a monodispersity as possible, while maintaining very high stability over time period of 30 days. The goal was achieved for two formulations, i.e., NE no. 10 and NE no. 11, which fulfilled the given requirements. Nonetheless, the best emulsion turned out to be NE no. 10 (the best solution: SCG AAS, concentration 0.1 mol/dm^3, fabricated under 1500 atm), which was later used in surface properties evaluation. In formulation optimization and other processes, it is common practice to make use of the answers produced by mathematical and statistical calculations using the design of experiments (DoE) and quality by design (QbD) approaches.

As is typical for RSM approaches, the ANOVA evaluation of the quadratic response surfaces predicted by a coordinate-exchange D-optimal plan for response variables Y_1 – Y_5 indicated that the quadratic model offered the best matching in every instance. [S1-S3]. The derived best-fit model had significant parameters, i.e., a negligible discrepancy between the experimental, adjusted, and forecasted R^2 coefficients, and a suitable number of degrees of freedom. All p-values for model fitting were less than 0.05, indicating that the terms and intercepts of the D-optimal model were significant for all dependent variables examined in this contribution. Table S5 summarizes the analysis of variance findings.

All three independent variables, i.e., concentration of AAS (A), homogenization pressure (B), and type of AAS (C), exhibited a two-factor interaction (2FI) and had the equivalent impact on the response Y_1 (particle diameter), both in individual and combined effects ((A), (B), (C), and (AB), (AC), and (BC)). Therefore, an appropriate combination of process parameters at desirable levels can ensure that the produced formulation will meet the criteria for an effective w/o nanoemulsion. From the point of view of response Y_2 (PDI), the main effect was observed also in terms of 2FI; however, in this case, concentration and type of AAS employed had the greatest influence on maintaining the lowest PDI values (both (A) and (B), as well as (AC). In the case of the response factors Y_3 and Y_4, the influence of independent variables (A) – (C) was exactly the same as for response Y_1. The exhibited a 2FI relationship with response factor, where all three process parameters had the same equivalent impact on the TSI values after 0 and 7 days, both individually and combined, i.e., (A), (B), and (C), as well as (AB), (AC), and (BC). Finally, for the response Y_5 (TSI after 30 days), only the linear influence of all three process parameters (A), (B), and (C) was observed, with equivalent impact (p-value: <0.0001).

Those results clearly demonstrate that the type of amino-acid based (AAS) surfactant and high homogenization pressure, in conjunction with the effect of the stabilizing agent concentration, ensure the formation of a stable w/o nanoemulsion that is exceptionally effective for the removal of graffiti coating. Therefore, appropriate selection of process parameters is crucial.

The polynomial regression equations that emerged from the ANOVA analysis after fitting the experimental values of the response factors with the D-optimal model were as follows:

Diameter (Y_1) = $+3.62 +0.9311A -2.57B +0.4048C[1] +1.50C[2] -0.9558C[3] -0.5726AB +0.2668AC[1] +0.6730AC[2] -0.4083AC[3] -0.0533BC[1] -0.7567BC[2] +0.6963BC[3]$ **(Eq. S2)**

PDI (Y_2) = $+0.0469 +0.0076A +0.0004B -0.0155C[1] +0.0340C[2] -0.0062C[3] -0.0039AB -0.0111AC[1] +0.0290AC[2] -0.0136AC[3] +0.0012BC[1] -0.0098BC[2] +0.0012BC[3]$ **(Eq. S3)**

TSI (0 days) (Y_3) = $+1.72 +0.5610A -0.4643B +0.4359C[1] +1.19C[2] +0.0123C[3] -0.2115AB +0.4481AC[1] +0.1991AC[2] -0.0952AC[3] +0.1743BC[1] -0.6030BC[2] +0.0537BC[3]$ **(Eq. S4)**

TSI (7 days) (Y_4) = $+12.88 +4.72A -4.61B -8.44C[1] +29.01C[2] -8.66C[3] -4.24AB -1.29AC[1] +8.94AC[2] -2.94AC[3] +3.31BC[1] -9.00BC[2] +3.72BC[3]$ **(Eq. S5)**

TSI (30 days) (Y_5) = $+16.02 +5.88A -5.04B -6.19C[1] +26.31C[2] -9.47C[3]$ **(Eq. S6)**

Table S5. ANOVA results for D-optimal randomized design quadratic model for dependent variables of graffiti remover w/o nanoemulsion formulation.

Source	Sum of sq.	Term df	F-value	p-value
dependent variable: diameter				
**Model**	258.96	12	52.08	< 0.0001
A- concentration	20.03	1	48.32	< 0.0001
B - pressure	164.74	1	397.54	< 0.0001
C – AAS type	31.91	3	25.67	< 0.0001
AB	5.95	1	14.35	0.0012
AC	5.66	3	4.55	0.0144
BC	6.42	3	5.17	0.0088
Lack of Fit	7.87	13		
S.D. = 0.6437, Mean = 3.43, R^2 = 0.9705, Adj. R^2 = 0.9519, Pred. R^2 = 0.8748				
dependent variable: PDI				
**Model**	0.0260	12	18.46	< 0.0001
A- concentration	0.0019	1	16.61	0.0006
B - pressure	3.311E-06	1	0.0282	0.8684
C – AAS type	0.0139	3	39.44	< 0.0001
AB	0.0003	1	2.33	0.1436
AC	0.0074	3	20.92	< 0.0001
BC	0.0009	3	2.59	0.0833
Lack of fit	0.0022	13		
S.D. = 0.0108, Mean = 0.0480, R^2 = 0.9210, Adj. R^2 = 0.8711, Pred. R^2 = 0.6761				
dependent variable: TSI (0 days)				
Model	57.54	12	65.73	< 0.0001
A - concentration	7.39	1	101.34	< 0.0001
B - pressure	5.31	1	72.75	< 0.0001
C - APGs	30.53	3	139.48	< 0.0001
AB	0.8110	1	11.12	0.0035
AC	3.04	3	13.88	< 0.0001
BC	3.16	3	14.46	< 0.0001
Lack of fit	1.39	13		
S.D. = 0.2701, Mean = 1.68 R^2 = 0.9765, Adj. R^2 = 0.9616, Pred. R^2 = 0.9127				
dependent variable: TSI (7 days)				
Model	12556.06	12	30.64	< 0.0001
A - concentration	559.76	1	16.39	0.0007
B - pressure	544.36	1	15.94	0.0008
C - APGs	8865.84	3	86.54	< 0.0001
AB	325.95	1	9.54	0.0060
AC	702.26	3	6.85	0.0026
BC	650.94	3	6.35	0.0036
Lack of fit	0.0000	6		
S.D. = 5.84 Mean = 13.07 R^2 = 0.9509, Adj. R^2 = 0.9198, Pred. R^2 = 0.7490				
dependent variable: TSI (30 days)				
Model	9606.87	5	17.55	< 0.0001
A - concentration	800.38	1	7.31	0.0119
B - pressure	639.40	1	5.84	0.0230
C - APGs	7430.44	3	22.63	< 0.0001
Lack of fit	0.0000	6		
S.D. = 10.46, Mean = 15.46, R^2 = 0.7714, Adj. R^2 = 0.7275, Pred. R^2 = 0.6455				

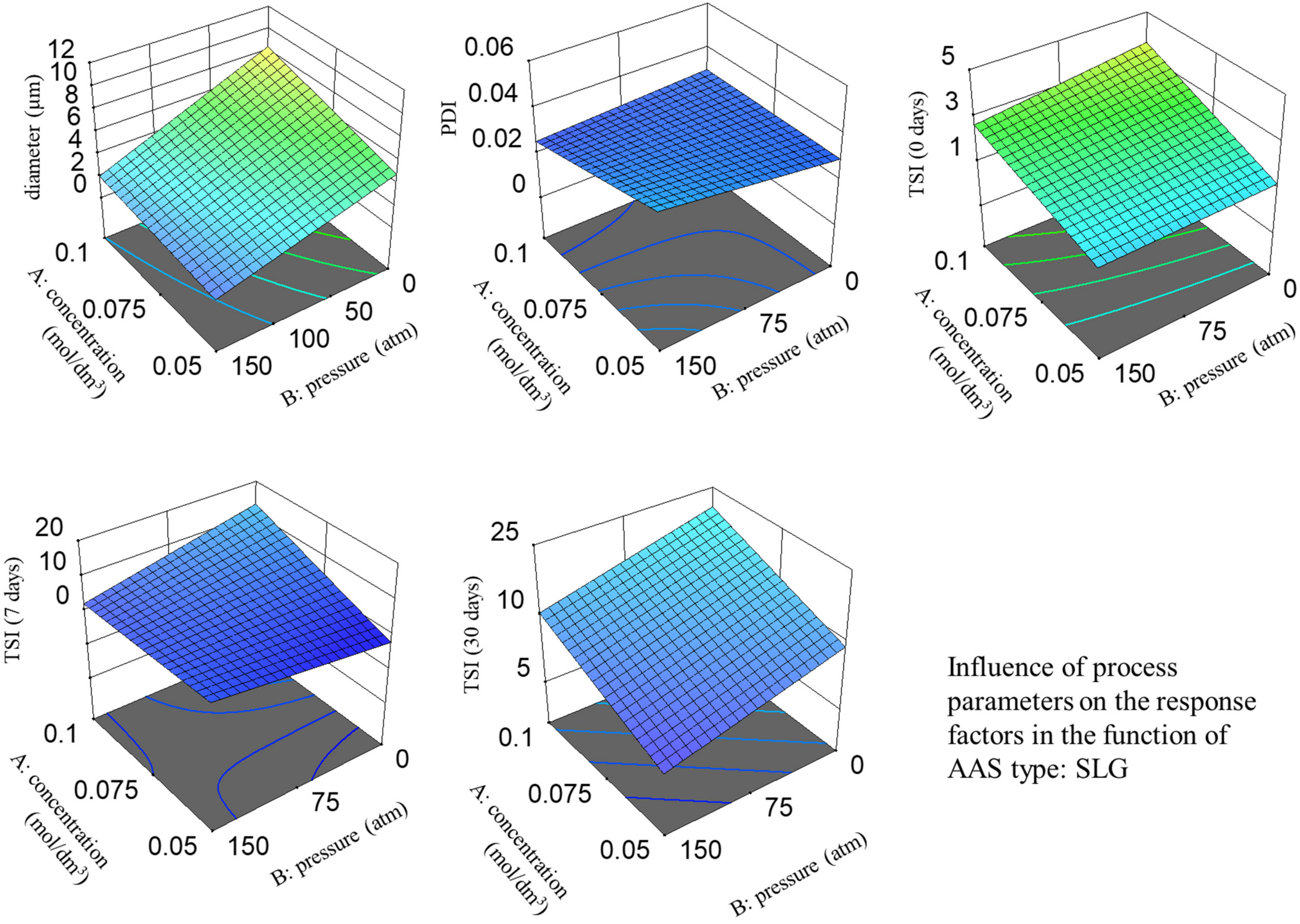

Figure S2. A graphical representation of the randomized quadratic *D*-optimal design response surfaces for the dependent variables Y_1 = diameter, Y_2 = PDI, Y_3 = TSI (0 days), Y_4 = TSI (7 days), and Y_5 (TSI 30 days) vs. independent variables (concentration of AAS (A), HPH pressure (B) as a function of AAS type used: SLG).

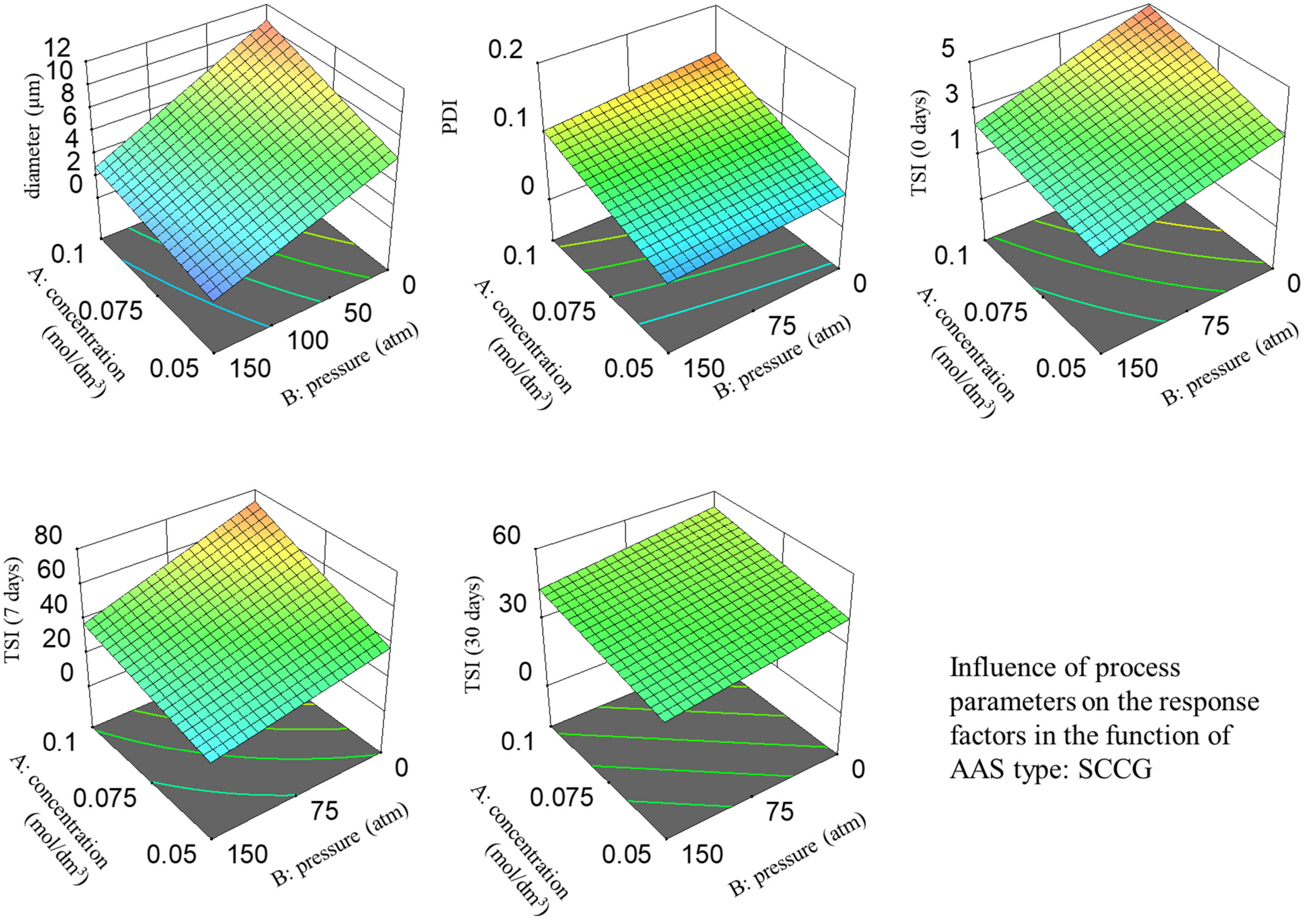

Figure S3. A graphical representation of the randomized quadratic *D*-optimal design response surfaces for the dependent variables Y_1 = diameter, Y_2 = PDI, Y_3 = TSI (0 days), Y_4 = TSI (7 days), and Y_5 (TSI 30 days) vs. independent variables (concentration of AAS (A), HPH pressure (B) as a function of AAS type used: SCCG).

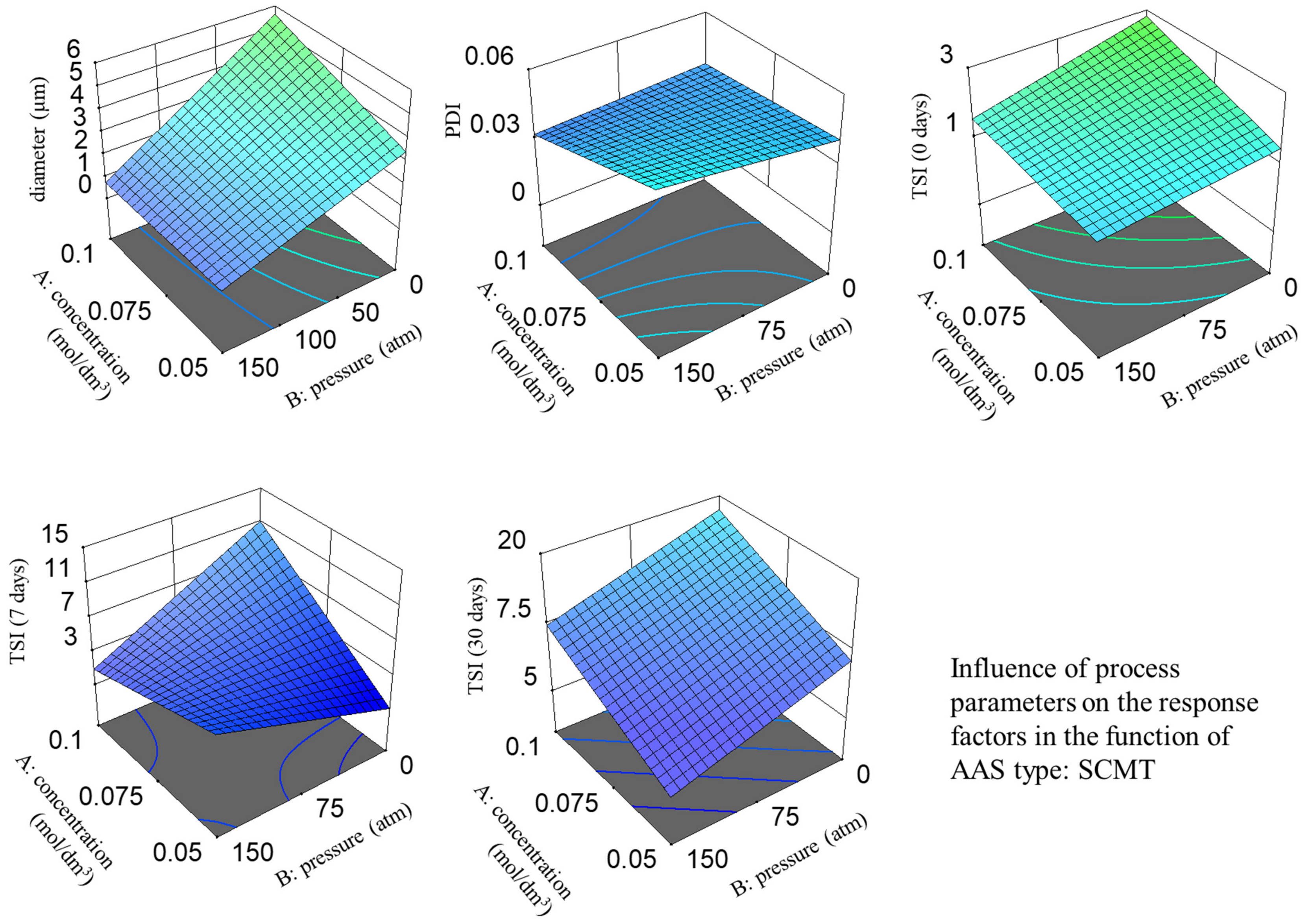

Figure S4. A graphical representation of the randomized quadratic *D*-optimal design response surfaces for the dependent variables Y_1 = diameter, Y_2 = PDI, Y_3 = TSI (0 days), Y_4 = TSI (7 days), and Y_5 (TSI 30 days) vs. independent variables (concentration of AAS (A), HPH pressure (B) as a function of AAS type used: SCMT).

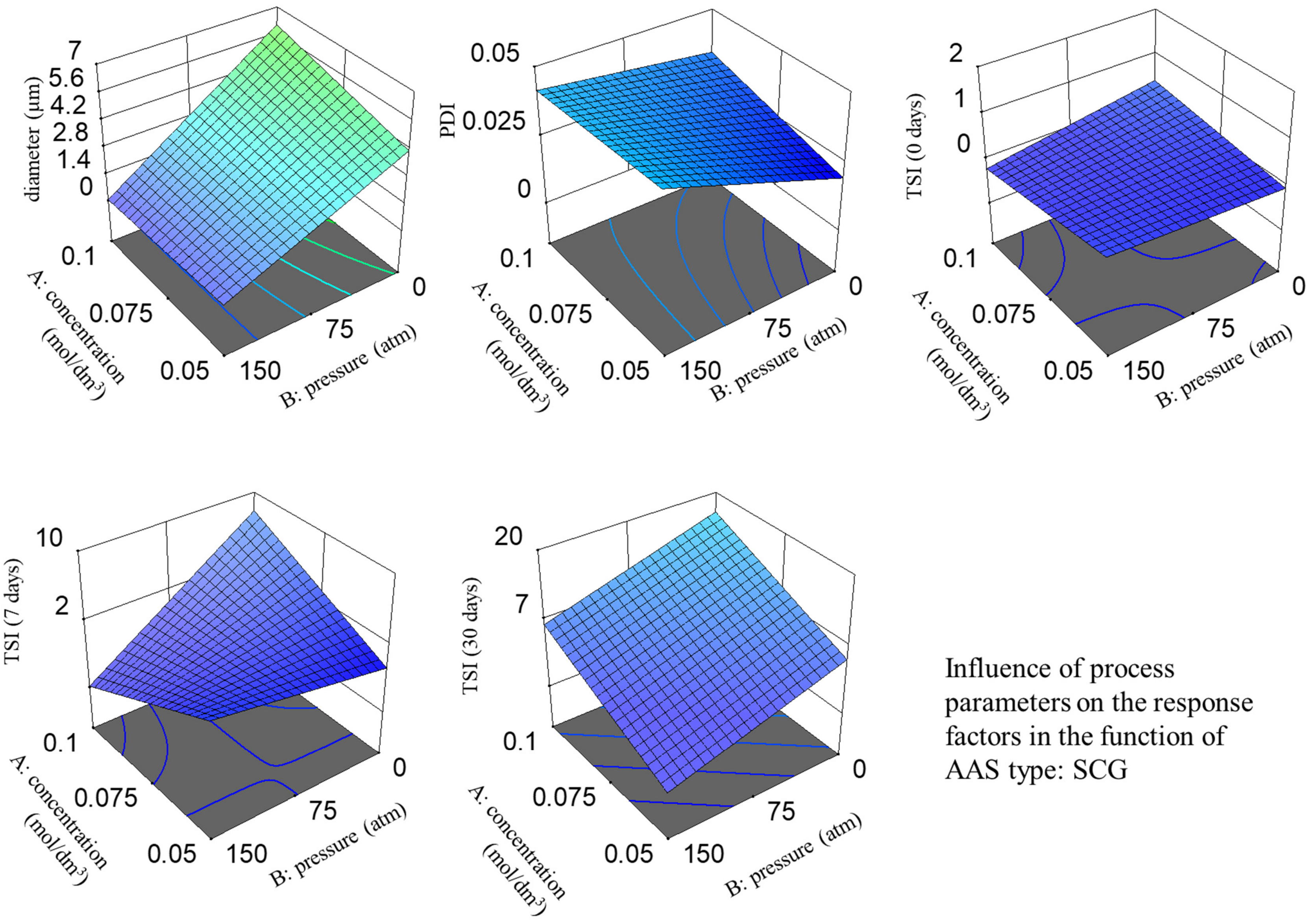

Figure S5. A graphical representation of the randomized quadratic *D*-optimal design response surfaces for the dependent variables Y_1 = diameter, Y_2 = PDI, Y_3 = TSI (0 days), Y_4 = TSI (7 days), and Y_5 (TSI 30 days) vs. independent variables (concentration of AAS (A), HPH pressure (B) as a function of AAS type used: SCG).

Bibliography

[S1] P. Khwanmuang, C. Naparswad, S. Archakunakorn, C. Waicharoen, C. Chitichotpanya, Optimization of in situ synthesis of Ag/PU nanocomposites using response surface methodology for self-disinfecting coatings, Prog. Org. Coat. 110 (2017) 104-113 https://doi.org/10.1016/j.porgcoat.2017.03.002

[S2] S. Balicki, Unit process optimization in the organic technology, Przem. Chem. 100 (2021) 490-497. https://doi.org/10.15199/62.2021.5.10

[S3] S. Balicki, I. Pawlaczyk-Graja, R. Gancarz, P. Capek, K.A Wilk, Optimization of Ultrasound Assisted Extraction of Functional Food Fiber from Canadian Horseweed (Erigeron canadensis L.), ACS Omega, 5(33) (2020) 20854-20862. https://doi.org/10.1021/acsomega.0c02181
